# Technical guidelines for head and neck cancer IMRT on behalf of the Italian association of radiation oncology - head and neck working group

**DOI:** 10.1186/s13014-014-0264-9

**Published:** 2014-12-29

**Authors:** Anna Merlotti, Daniela Alterio, Riccardo Vigna-Taglianti, Alessandro Muraglia, Luciana Lastrucci, Roberto Manzo, Giuseppina Gambaro, Orietta Caspiani, Francesco Miccichè, Francesco Deodato, Stefano Pergolizzi, Pierfrancesco Franco, Renzo Corvò, Elvio G Russi, Giuseppe Sanguineti

**Affiliations:** Radioterapia AO Ospedale di Circolo-Busto Arsizio (VA), Piazzale Professor G. Solaro, 3, 21052 Busto Arsizio, VA Italy; Radioterapia IEO-Milano, Milan, Italy; Radioterapia Az. Ospedaliera S. Croce e Carle-Cuneo, via M. Coppino 26 12100, Cuneo, Italy; Radioterapia Arcispedale S. Maria Nuova AO-Reggio Emilia, Emilia, Italy; Radioterapia PO S.Donato-AUSL-Arezzo, Arezzo, Italy; Radioterapia Azienda Ospedaliera ASL Napoli 1-Napoli, Napoli, Italy; Radioterapia A.O.U. Maggiore della Carità-Novara, Novara, Italy; Radioterapia Ospedale Fatebenefratelli, Isola Tiberina-Roma, Roma, Italy; Radioterapia Policlinico Universitario ‘A.Gemelli’-Roma, Roma, Italy; Radioterapia Università Cattolica del S. Cuore -Campobasso, Roma, Italy; Dipartimento SBIMOF Sezione di Scienze Radiologiche, Università di Messina, Piazza Pugliatti Salvatore, 1, 98122 Messina, ME Italy; Dipartimento di Oncologia, Radioterapia Oncologica, Università di Torino, Turin, Italy; Oncologia Radioterapica, IRCS S. Martino-IST- Istituto Nazionale per la Ricerca sul Cancro, Università Genova, Genova, Italy; UOC Radioterapia Istituto Tumori Regina Elena Roma (RM), Roma, Italy

## Abstract

Performing intensity-modulated radiotherapy (IMRT) on head and neck cancer patients (HNCPs) requires robust training and experience. Thus, in 2011, the Head and Neck Cancer Working Group (HNCWG) of the Italian Association of Radiation Oncology (AIRO) organized a study group with the aim to run a literature review to outline clinical practice recommendations, to suggest technical solutions and to advise target volumes and doses selection for head and neck cancer IMRT. The main purpose was therefore to standardize the technical approach of radiation oncologists in this context. The following paper describes the results of this working group. Volumes, techniques/strategies and dosage were summarized for each head-and-neck site and subsite according to international guidelines or after reaching a consensus in case of weak literature evidence.

## Introduction

Performing intensity-modulated radiotherapy (IMRT) in head and neck cancer patients (HNCPs) requires training [1] and experience. For example, in the 02–02 Trans Tasman Radiation Oncology Group (TROG) trial, comparing cisplatin (P) and radiotherapy (RT) with or without tirapazamine, a major quality defect of the irradiation approach in terms of dose and target volume selection and delineation was found in 12% of patients and was associated with a 24% lower loco-regional control rate at 2 years [2]. Hence, in 2011, the Head and Neck Cancer Working Group (HNCWG) of the Italian Association of Radiation Oncology (AIRO) organized a study group to outline clinical practice recommendations regarding techniques, treatment volumes and doses to be employed during head and neck IMRT. The main purpose was to standardize technical approaches of professionals participating into AIRO head and neck cancer trials.

## Material and methods

The first participants (AM, DA, AM, LL, RM, GG, OC, FM, FD and RC) were chosen on a voluntary basis among the HNCWG members. The group was coordinated by an expert head and neck radiation oncologist (RC). Each member was in charge of a specific topic. At the end of the first draft (February 2012) the whole document was reviewed by all the HNCWG members in order to discuss critical issues and to homogenize the manuscript structure. The revised draft was again reviewed by 5 radiation oncologists with particular expertise in head and neck IMRT (RVT, PF, SP, ER and GS).

### General aspects

#### Treatment strategy

RT is one of the mainstay treatment options for head and neck cancers (HNCs), along with chemotherapy (ChT) and surgery (S). In general, RT ± ChT is the preferred approach for head and neck squamous cell carcinomas (HN-SCC) whenever organ preservation is desired or the tumor is unresectable at presentation (cT4b) or the patient is considered not amenable to surgery. For early T-stage lesions (cT1 and selected cT2) without lymph nodes involvement (cN0), treatment should be monomodal (RT vs S), while for locally advanced disease (cT3-cT4 or any T,cN+), treatment is usually multimodal (S followed by RT ± ChT vs RT and concomitant ChT ± S). Concomitant ChT (usually platinum-based) has been shown to increase overall survival (OS) in stage III and IV disease over RT alone [3]. Recently published long-term results of the RTOG 91–11 trial in larynx cancer failed to show such an advantage over sequential ChT-RT because of an increased number of non-cancer-related deaths in the concomitant arm, suggesting a higher long-term toxicity rate for ChT-RT [4]. Induction and adjuvant ChT are less effective than concomitant ChT [3,5]. However, within an organ preservation strategy, induction ChT may become an option to select responders who might potentially benefit from an organ preservation approach, driving eventual further treatments (S vs RT) on the basis of response to neoadjuvant ChT [6,7]. Recent published trials found no benefit from the addition of Taxotere(T)-Platinum (P)-5Fluorouracil(F) induction ChT to concomitant ChT/RT or cetuximab-RT compared to concomitant ChT/RT or RT alone [8-10]. Another alternative to concomitant ChT is cetuximab, an epidermal growth factor receptor inhibitor, which, combined to RT, has been shown to provide a better OS than RT alone in HN-SCC [11]. However, Erbitux has never been tested against the standard treatment (concomitant platinum-based ChT-RT) and consequently its role is somewhat unclear. Moreover, when Erbitux was added to concomitant ChT-RT, no additional benefit was found [12]. After concomitant ChT-RT, S is usually reserved for eventual locoregional disease persistence. Regarding neck management, most Institutions nowadays use FDG-PET/CT at 10–12 weeks to select patients at risk of for residual nodal disease [13]. The decision to add adjuvant treatments after upfront surgery is made once the pathology report is available. RT is usually added for high risk features including positive resection margins (PRMs), advanced T stage (selected pT3 and all pT4), perineural invasion, lymph-vascular invasion, any nodal stage higher than pN1, and extra-capsular lymph-nodal extension (ECE); ChT is added for PRM and ECE. Therefore, treatment is usually multidisciplinary and any decision should be discussed within the tumor board involving all specialties. Aspects such as nutrition, oral care and restoration/preservation of swallowing and phonatory function (speech therapy) are also important and should be considered whenever feasible.

### IMRT (Intensity-Modulated Radiation Therapy)

**Figure 1:**
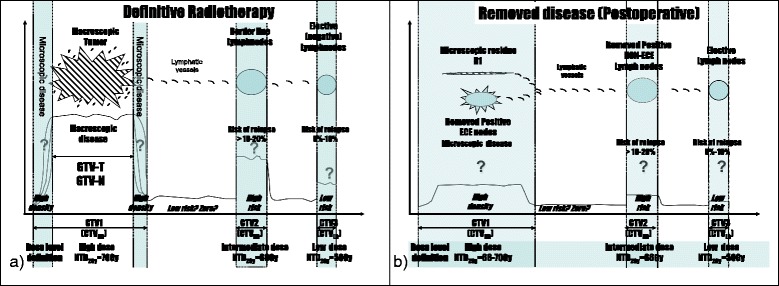
**Volumes at risk in HNC (a) Definitive and b) postoperative RT).** The Question mark "?" refers to the uncertainty of the tumour-cell density.

Though few Institutions pioneered IMRT for HN-SCC during mid-90s, this technique has become widespread during the last decade. The aim of IMRT is to achieve more conformal dose distribution over standard 3D conformal RT (3DCRT) and this in turn allows for better sparing of normal structures (i.e. parotid glands). This potentially translates into fewer late side effects (xerostomia) and improved quality of life [14]. With IMRT, the physician identifies the target volumes(s) and the organs at risk (OARs) appropriate for a given clinical condition. The dose to the target is usually proportional to the estimated tumour burden (Table 1 and Figure 1).

**Table 1:** **Volumes at risk in HNC**

**Burden of disease**	**Description**	**ICRU definition**	**Adopted definition**	**Finality**	**T – level**	**N – level**	**Dose level definition**	**Dose level NTD**	**Solution examples (Total-dose Gy/single-fraction Gy/numbers of fractions**			
									**Definitive**		**Postoperative**	
									**Conventional**	**Slightly accelerated**	**Conventional**	**Slightly accelerated**
Macroscopic	Known gross disease	GTV	GTV	Definitive	Primary tumor	Each Positive-nodes	High Dose	70 Gy	70/2/35[18]	70/2.12/33[20]		
High risk of microscopic disease	Risk of relapse > 10-20% [54,55]	CTV	CTV_HD_^	Definitive	Peri-GTV areas considered to contain potential microscopic disease [38] (T + 10 mm)*	Positive Nodes + 5[41]-10[53]mm				66/2.2/30[17,18]		
			(CTV1)							65/2.17/30[77]		
				Post-S	Surgical bed with soft tissue involvement (Positive or close margins): PTB +0.5-1 cm according to anatomical barriers [41].	Nodal region with extracapsular extension [41]: PTB plus 1 cm up to the skin [56].^ç^		66-70 Gy			≥63/1.8/35[78]	65/2.17/30[77]
												66/2/33[79]
			CTV_HR_	Definitive	Preferential areas of diffusion.(Optional) [56]	Border-line lymph-nodes [51,57]	Intermediate Dose	60 Gy	63/1.8/35[18]	60/2/30[17,18]		
			(CTV2)	Post-S	Surgical bed without soft tissue involvement [41]	Nodal region without extracapsular extension		66§ Gy		59.4/1.8/33[20]	≥63/1.8/35[78]	
Low risk of microscopic disease	Risk of relapse 5-10% [41,80]	CTV	CTV_LR_	Definitive	Structure or compartment adjacent to tumor [56]	Elective nodal regions, defined for each primary-tumor subsite#	Low dose	50 Gy	58.1/1.66/35[18]	54/1.8/30[17,18,77]		54/2/27[79]
			(CTV3)	Post-S				50 Gy		50.4/1.8/28[20]	57.6/1.8/32[78]	54/1.8/30[77]

*Depending from anatomic barrier;§ though one prospective study failed to show a benefit for 66 Gy over 60 Gy in the high risk post-operative region [78]*, this is the dose recommended by some cooperative groups (EORTC* [79]*); PTB: postoperative tumour bed; ^ definition of the high-risk region is controversial* [18] *; D = Definitive RT; Post-S = postoperative.* CTV_HD_ *: High Disease;* CTV_HR=_ *High Risk; LR = Low risk.* ^*Ç*^ *in case of muscular infiltration (i.e. sternocleidomastoid muscle) at least the portion of the muscle surrounding the node* [47] *should be included. # Similarly, it would be appropriate to include the whole muscle (i.e. sternocleidomastoid muscle) in CTV3/LR or when grossly infiltrated at some level.*

It should be noted that the maximum prescribed dose has empirically evolved as the highest tolerated dose taking into account the surrounding normal structures. A ‘definitive’ treatment typically includes 2–3 dose levels within the same patient. Using IMRT a different dose to different targets can be delivered with sequential plans (as for 3DCRT) or with a simultaneous integrated boost (SIB). It has been shown that the latter approach provides better dose conformity compared to several consecutive plans [15]. When a single plan is prescribed, CTV1 receives both a higher total dose and a higher dose/fraction (d./f.) compared to the other CTVs. This results in an even higher biologically equivalent dose (BED) compared to other CTVs. However, since all dose levels are delivered throughout the same number of fractions, targets must receive different fractionations.

Two solutions have been proposed [16] (see Table 1). The first approach is to maintain conventionally fractionated doses to the highest dose CTV (1.8-2 Gy per fraction) (Table 1) whereas the elective targets receive a lower d./f. (1.6-1.8 Gy). The choice of the latter is made taking into consideration the normalized total dose in 2 Gy fractions (NTD). The second strategy is to deliver a slightly hypofractionated dose to the GTV and standard fraction doses to the CTVs [17]. Each strategy has pros and cons and no universally accepted schedule has been developed. The former solution may lead to the use of a very low d./f. according to current standards; a daily d./f. below 1.6 Gy should be avoided. Notably, in one study, 58.1 Gy in 35 fractions were able to control the microscopic low-risk disease [18]. The latter approach has the disadvantage of using a higher d./f. (up to 2.5 Gy) than the standard fractionation for the GTV potentially exposing normal tissues embedded within the high dose CTV to the risk of increased late toxicity. Moreover, this results in a higher than standard weekly total dose to normal tissues (i.e. mucosa) within the high dose region. According to some authors [19], 66 Gy/30 f. (2.2 Gy) should not be used in the context of concomitant CT. On the other hand, a d./f. up to 2.12 Gy is used in the context of concomitant CT (P, 100 mg/m2 x 3 cycles) for nasopharyngeal cancer [20]. Recently some authors have reported initial experience in advanced HNC with a d./f. up to 2.25 Gy in 30 fractions concomitantly to CT. This schedule seems feasible with an acceptable but not negligible acute toxicity rate, while a longer follow-up is needed to fully evaluate late effects [21,22]. In conclusion, hereinafter we will refer to IMRT as a SIB approach. The choice of the fractionation schedule (d./f.) is left to each Institution taking into account the aforementioned considerations. Whenever available, we will refer, for each primary site, to the fractionation schedules that have been tested clinically. We encourage each Institution to follow patients and investigate its own pattern of failure.

### Practical implications of induction/concomitant CT

HNCs are typically treated once a day, i.e. 5 fractions per week (f/w) (standard fractionation). Although a few studies, and one meta-analysis, have shown better loco-regional control rates, and even improved survival, when multiple fractions per day are delivered [23-25], it is doubtful whether the treatment acceleration (more than 5 f/w) is beneficial also in the context of concomitant CT. Two randomized studies [26,27] failed to show an advantage of concomitant chemo/accelerated-RT over standard CT/RT. Moreover, there are practical limitations in the number of treatment sessions that can be delivered per day with IMRT due to its workload intensiveness (each treatment session typically takes more than 15 minutes). RTOG [26] adopted a mildly accelerated radiation schedule (6 f/w) with concomitant CT as a standard, delivering the 6th fraction as a second daily dose on Friday (at least with a 6 hour gap) or on Saturday. In the context of concomitant CT there are no clinical data showing the benefit of multiple fractions per day (or more than 5 f/w) or a single fraction per day higher than 2 Gy.

Regarding induction CT, one potential advantage/pitfall is the potential downsizing of the primary tumour and lymph nodes. This can be successfully exploited in selected situations where the delivery of the highest dose level is limited by the tolerance of the surrounding normal structures (i.e. optic pathways for T4 NPC) [28]. On the other hand, it has been recommended that the *initial* rather than the *post*-chemo volume should encompass the high-dose region during contouring and planning [29]. With this in mind, the planning session should be carried out both before and after induction CT, if possible using the same immobilization device.

### Practical implications of upfront surgery

Extensive surgery can disrupt normal anatomy and violate tissues that generally are not considered at risk of microscopic disease. In general, the whole operative bed is considered at some/low risk after surgery, with the initial site of disease at highest risk. This implies that, when a flap reconstruction is performed, the whole flap should be included at least in the lowest dose volume. Regarding the whole operative bed, doses in the order of 50–54 Gy (2 Gy per fr) have been used [30,31]. It should be noted that the ‘classical’ recommendation is to include any surgically violated sites regardless of final pathology (i.e. the dissected neck that is found to be negative, pN0, should still be included in the lowest dose level) (Table 1). Regarding the initial site of disease, its identification is best based on pre-surgery imaging studies and operative notes. When incomplete resection is suspected, a repeated PET/CT at > 1 month after surgery has been shown to detect macroscopic residual disease in a significant proportion of patients [32]. Postoperative treatment should be started as soon as healing takes place (usually 2 weeks) and not later than 6–8 weeks after surgery [31]. Patients who fail after surgery may have a peculiar lymphnode-invasion pattern due to the distortion of normal lymphatic flow. Therefore, in this setting, lymph node involvement may be less predictable than in the previously untreated setting and hence deviate from the recommendations here provided.

### Pre-treatment evaluation

The following procedures are recommended before starting RT:

1. Biopsy of the primary lesion. Fine needle aspiration (or more aggressive procedures) of suspicious lymph nodes may also be an option when the primary lesion is not evident (cTx) or when the presence of positive lymph nodes may have prognostic and/or therapeutic implications (i.e. definition of treatment volumes or the addition of CT).
2. Panendoscopy under general anaesthesia with ‘blind’ tongue-base, tonsillar fossa, pyriform sinuses, Rosenmuller fossa biopsies or unilateral/bilateral tonsillectomy in unknown primary tumours.
3. Anamnesis (including tobacco and alcohol use, sexual habits, and current medications) and medical examination (including weight and performance status evaluation).
4. Indirect laryngoscopy and flexible endoscopic examination.
5. Complete dental evaluation (except for those receiving narrow fields for larynx cancer). Any required dental extractions must be perfomed and fluoride prophylaxis instituted prior to RT.
6. Speech pathology evaluation, including instrumental and/or clinical swallowing assessment and administration of pretreatment swallowing and trismus exercises, completion of pretreatment QoL questionnaires.
7. Nutritional evaluation.
8. Completion of the following laboratory studies: CBC (complete blood count), metabolic panel including: sodium, potassium, glucose, calcium, magnesium, BUN (blood urea nitrogen), serum creatinine, total protein, albumin, alkaline phosphatase, total bilirubin, AST, ALT, creatinine clearance, and thyroid function (TSH).
9. Completion of the following radiological studies:
  9.1 Head-and-neck CT with <3 mm contiguous slices *(with contrast enhancement, unless contraindicated*);
  9.2 Whole body ^18^FDG-PET/CT scan for locally advanced disease and for unknown primary sites (integration of both high resolution contrast-enhanced CT and dedicated PET acquisition through the head and neck region are strongly suggested; as an alternative, CT of the chest ± abdomen, in particular for salivary gland tumours);
  9.3 Head-and-neck MRI with gadolinium including T1- and T2-weighted sequences in at least 2 different planes (strongly suggested for tumours involving the tongue base, salivary glands and nasopharyngeal cancer).
10. Audiogram is recommended if the inner ear is to be irradiated at mean dose ≥ 40 Gy.
11. Post-operative MRI/CT, pre and post-operative PET (optional);
12. Testing the tumour specimen for Human Papilloma Virus (HPV) is also strongly recommended via in-situ hybridization or, indirectly, via p16 IHC (Immunohistochemistry) for oropharyngeal cancer, WHO-Type 1 nasopharyngeal cancer, and unknown primary tumours [33]. Testing the tumour specimen for Epstein Barr Virus (EBV) is strongly recommended for both nasopharyngeal and unknown primary tumours.

### Simulation

1. The patient is usually set up on the treatment table in supine position. The immobilization device is generally a thermoplastic mask immobilizing both head and shoulders [34]. A mouth piece is also indicated for all non-edentulous patients. A tongue depressor is generally not recommended except for tumours of the nasal cavity and paranasal sinuses (to move the tongue and the mouth floor away from the irradiated volume) or for tumours of the bottom of the oral cavity (to displace the upper part of the oral cavity). Dental prostheses have to be removed. The position of the head is usually neutral and comfortable except in selected situations detailed in each section. The patient is instructed to breath normally/quietly and not to swallow during scanning. Scars are usually wired. A 3–5 mm bolus over the larynx is also optional depending on the beam energy and the patient’s thickness when the larynx is part of the target (especially for lesions involving the anterior commissure and beyond). A 3–5 mm bolus is also to be considered over areas of skin infiltration and/or after surgery in case of nodal extra-capsular extension (ECE).
2. Treatment planning CT scan should be performed having the patient in the treatment position. I.V. contrast liquid administration at the time of simulation is also recommended. MRI-scan planning is optional.
3. CT-scan thickness should be 0.3 cm or less through the region that contains the primary target volumes. The regions above and below the target volume may be scanned with slice thickness up to 0.5 cm. MRI and PET/CT scans may be included to assist in defining target volumes as appropriate.
4. The GTV, CTV and PTV and normal tissues are outlined on all the appropriate CT slices.
5. Patients should be trained to avoid swallowing in order to reduce organ motion during treatment.

### Volume and ICRU reference point definitions

The definition of volumes is in accordance with ICRU Reports n° 50 [35] and n° 83 [36]:

1. The **Gross Tumour Volume (GTV)** is defined as all known gross disease determined by CT, clinical information, endoscopic findings and MRI in case of tumours treated after biopsy alone. Functional information can be used to define sub-GTVs that are to receive some additional absorbed dose [37].
  Typically, different GTVs are defined for the **primary tumour (GTV-T)** and **the regional node(s) (GTV-N)**. Yet in those clinical situations in which the metastatic node cannot be distinguished from the primary tumour, a single GTV encompassing both the primary tumour and the node(s) may be contoured (GTV-TN) [38].
  Furthermore, in consideration of adaptive RT, any changes occurring within the GTV during treatment can be quantified with anatomic- and/or functional-imaging techniques, allowing for the definition of a modified GTV in order to adjust the absorbed-dose distribution [39,40].
  Thus, because GTV contouring can vary according to the diagnostic modality used (e.g. clinical examination, anatomic and/or functional imaging) and the time/dose of acquisition with respect to the start of treatment (e.g. in the case of adaptive RT), a clear annotation is required. For example, ICRU 83 recommends specifying the imaging technique and pre-delivered dose: e.g. GTV-T (MRI-T2, 30 Gy), GTV-T (clin, 0 Gy).

2. The **Clinical Target Volume (CTV)** is contoured by the treating physician and is defined as a volume of tissue that contains a demonstrable GTV and/or subclinical malignant disease with a certain probability of occurrence considered relevant for therapy [38]. The notion of subclinical malignant disease includes the microscopic tumour spread at the boundary of the primary-tumour GTV, the possible regional infiltration into lymph nodes, and the potential metastatic involvement of other tissues. The selection of the tissues that bear risk for microscopic infiltration outside the GTV is a probabilistic assessment integrating the biological and clinical behaviour of the various tumour entities. Consideration may also be given to the presence of any specially radiosensitive normal tissue (Organs at Risk) as well as to other factors such as patient’s general conditions [35].
  2.1. Clinical experience indicates that in the region around GTV (Figure 1 a,b) there is generally subclinical involvement, i.e. individual malignant cells, small cell clusters, or microextensions, which cannot be detected through clinical staging procedures. The GTV together with this surrounding volume of local subclinical involvement can be defined as a clinical target volume **(CTV-T for primary tumour, and CTV-N for metastatic lymphadenopaties, etc.)**. If the same dose is prescribed for two such CTVs and if they are close to each other, they can be labelled CTV-TN. This volume has to be considered for therapy and, if included, should be irradiated adequately to achieve cure. The margin between each GTV and its CTV should be typically 10–20 mm, with a minimum of 5 mm except in those areas where the GTV is immediately adjacent to structures known to be uninvolved (i.e. anatomic barriers). To account for the risk of extracapsular spread, a margin should also be added to the involved nodes [41].
  2.2. Additional volumes (CTVs) with presumed subclinical spread (Figure 1a,b) (e.g. regional lymph nodes, N0) at a distance from a GTV also need to be considered for therapy [42]. In this situation the prescription is based on the assumption that in some anatomically definable tissues/organs, there may be cancer cells at a given probability level, even though they cannot be detected as they are subclinical. The level of probability is based on clinical experience from adequately documented treatments and follow-up. For the purpose of treatment prescription, it can usually be described in terms of frequency of risk for later detectable manifestations (failure rate), when untreated adequately in the subclinical setting [43]. There is no general consensus on which probability is considered relevant for therapy [18,38,44], but typically *a probability of occult disease higher than 5-10% is assumed to require treatment*. However, the volume could be defined as at ***“high-risk” (CTVHR or CTV2)*** or ***“low-risk” (CTVLR or CTV3)*** (Figure 1a/b) in consideration of the probability and entity of microscopic infiltration and could be targeted at different dose levels [18,41,44].
    Moreover, in HNC the probability of pathologic lymph-node involvement has been well described, and the distribution follows a predictable pattern allowing clinicians to tailor the CTV to the primary-tumour location and extent [18,45-47].
    In postoperative cases, the CTV includes the operative bed and adequate margins according to an assessment of the risk of subclinical disease.
    The contouring of GTV and CTV are based on purely anatomic-topographic and biological considerations (morphology, such as ulcerative or exophytic, infiltrative or pushing front) with no regard to technical treatment factors [35,43]. Consequently, rather than simply expanding the GTV uniformly, it is recommended that the CTV should be contoured on a slice-by-slice basis [48].
    Hereinafter, peri-GTV, CTVHR and CTVLR will be respectively defined CTV1 (e.g. CTV-T 1; CTV-N 1; CTV-TN 1), CTV2 and CTV3.

3. The Planning Target Volume (PTV) will provide a margin around each CTV to compensate for the uncertainties of treatment set-up and tissue deformation. An isotropic expansion of 5 mm is typically added around the CTV to define each respective PTV. For patients undergoing daily IGRT, the margins are sometimes cut down to 3 mm, though this is controversial [49]. Margins can be further expanded in special situations (i.e. concerns on set-up error). Nevertheless, institution specific margins are strongly recommended.
4. In patients who receive neoadjuvant CT, the post-CT GTV will differ from what is seen on diagnostic images and the former should be outlined. The CTV, however, should be tailored to encompass the preCT GTV [29] (see Figure 2).

**Figure 2:**
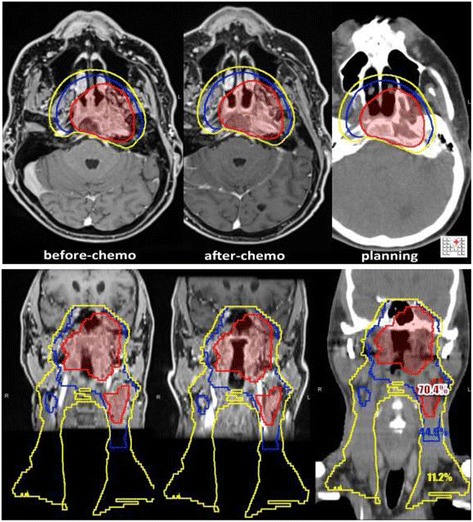
**Nasopharynx.** Countered volumes (before and after ChT).
5. To facilitate planning a suffix indicating the prescribed dose to each CTV should be used, e.g. CTV70, CTV60, CTV50, etc.
6. Unless otherwise specified, here we will refer to the 3 definitive and 2 postoperative dose levels as reported in Table 1.

### Primary-tumour contouring (GTV-T): high dose volume (CTV-T 1)

The GTV is usually defined based on physical examination and imaging findings. While the addition of MRI to CT contributes to improve tumour detection and observer consistency, the role of functional imaging to define the GTV is less established. Some authors disregard the information from FDG PET-CT [50].

Once identified, the GTV is usually expanded to its corresponding CTV (CTV1) by 0–10 mm depending on the presence of anatomic barriers, image quality and the presence of artefacts (dentures). With 3DCRT the whole portion of the sub-site, or a major part of it, is included in the CTV1 (e.g. the whole larynx and the whole rinopharynx in the case respectively of laryngeal and nasopharyngeal primaries). Conversely, with IMRT and according to ICRU directions, there is the tendency to limit the high dose region to the part of the sub-site, involved by disease.

### Positive neck nodes (GTV-N): high dose volume (CTV-N 1)

Lymph node stations or levels in the neck follow the surgical nomenclature. Cervical nodes are usually considered positive on contrast-enhanced CT scans if they show one or more of the following features: maximum axial diameter > 1 cm (5 mm if retropharyngeal); oval/round as opposed to reniform shape; suspected focal necrotic hypodensity; irregular enhancement pattern; presence of extra-capsular penetration as judged by spiculated margins [51] and clusters of 3 or more borderline nodes [52]. A station is considered positive if it contains positive lymph nodes. For positive nodes, a ‘full’ radiation dose is usually recommended. However, in this case, only the positive lymph node(s) and not the whole station is (are) contoured as GTV. Moreover, expansion from GTV to CTV1 does not imply that the whole level would be part of CTV1. Some authors would add 5 [41]-10 [53]mm around the GTV of lymph nodes to account for potential subclinical (extracapsular) spread [41]*.*

### Intermediate dose volume: high risk volume (CTV 2)

Within a definitive treatment, the definition of the intermediate dose region is highly subjective, variable across Institutions [18] and considered optional. Ideally it would correspond to the regions at high risk of containing microscopic disease (>10-20% [54,55]), preferential regions of diffusion around the primary tumour [56] or lymph nodes that are borderline or suspicious. (i.e. between 7–9 mm in greatest axial dimension in neck levels III through IV; with a rounded appearance defined as a width to length ratio greater than 0.5; lack of fatty hilum or a cluster of three or more borderline nodes) [51,57]. The factors affecting this risk should be taken into account for each case: tumour stage, size, thickness (3 mm or more is associated with a high metastatic risk for oral cavity tumours), differentiation, keratinization status, lymphatic vessel invasion in the tumour specimen, and whether other neck levels are involved [16].

Again, as for positive nodes, it is unclear whether the whole level at risk should be included within a given higher dose or only the suspicious node. We usually favour the latter, since in one study all neck failures originated from pre-treatment suspicious nodes [18].

### Elective neck contouring: low risk volume (CTV 3)

There are several definitions available for neck nodal levels and here we will refer to the one used by cooperative groups (RTOG/EORTC). Accordingly, there are several papers and atlases that have reported guidelines for nodal level contouring both without and after surgery [47,58,59]. Compared to volumes treated with 3DCRT, current definitions of neck levels provide only partially overlapping volumes and the mismatch is particularly evident for some levels (i.e. levels IB and V) [59]. However, once identified, it is possible to irradiate precisely and selectively different node levels wit IMRT. Hereafter we will refer to the neck nodal levels ipsi- and contra-lateral to the primary site for each subsite.

*Finally, it would be appropriate to include the whole muscle (i.e. sternocleidomastoid muscle) in CTV3/LR or when grossly infiltrated at some level.*

### Organ at risk (OAR)

#### Definition

Several OARs and tissues are routinely contoured on each patient planning CT as follows: mandible/TM joints, brain, brainstem, cord, parotid glands, upper gastrointestinal mucosa, larynx, oesophagus, brachial plexuses, inner ears, optic chiasm, optic nerves, ocular bulbs, lenses, major lacrimal glands, pituitary gland, submental connective tissue. For planning purposes, the cord and brainstem are usually expanded (by 5 mm) as well as the optic pathway structures (optic chiasm, optic nerves and both retinas) to form a PRV (planning reference volume). PRV expansion, as PTV expansion, depends on set-up accuracy and set-up errors as well as on the IGRT technique.

Optional (that are usually not constrained) OARs include: submandibular glands, thyroid gland, masticatory spaces, upper, middle, and inferior constrictor muscles, crico-pharyngeal muscle, carotid arteries, lung apexes.

### OARs dose (volume) objectives

Dose volume objectives are summarized in the Table 2.

**Table 2:** **Organs at risk**

**OAR** **[** 81 **]**	**Priority**	**Endpoint**	**Goal**	**Minor variation**	**Comment**
Cord	PRIM	0.1 cc	Dmax ≤ 44-45 Gy	Dmax 46 Gy	
Cord (PRV)	PRIM	0.1 cc	Dmax 44–48 Gy	Dmax 48–50 Gy	
Brain	PRIM	1 cc	Dmax 60 Gy	Dmax 63 Gy	
Temporal lobes	PRIM	1 cc	Dmax 60 Gy	Dmax 65 Gy	
Brainstem (PRV)	PRIM	0.1 cc	Dmax 54 Gy	Dmax 60 Gy	
Chiasm (PRV)	PRIM	0.1 cc	Dmax 54 Gy	Dmax 60 Gy	
Optic nerve (PRV)	PRIM	0.1 cc	Dmax 54 Gy	Dmax 60 Gy	
Larynx	PRIM	1 cc	Dmax 73.5 Gy	Dmax 77 Gy	
Mandible	PRIM	1 cc	Dmax 70–73.5 Gy	Dmax 75–77 Gy	
Inner ear	SEC	D mean	<50 Gy	<52.5 Gy	
Larynx (without cartilaginous framework)	SEC	V50	<25%	<30%	Oedema
Larynx (supraglottis)	SEC	Dmax	<66 Gy		Dysphonia
Larynx (whole organ)	SEC	Dmax	<50 Gy		Aspiration
Mandible	SEC	V55	<20%		
Esophagus	SEC	1 cc	Dmax 45 Gy	Dmax 55 Gy	
Parotid gland	SEC	V30	<50%	<60%	at least one
	SEC	Dmean	≤26 Gy		at least one
	SEC	V40	<33% (contralat)		
Upper GI mucosa (outside PTV)	SEC	1 cc	<30 Gy	<36 Gy	
Upper GI mucosa (whole volume)	SEC	V66.5	Dmax 64 Gy (<3 %?)	Dmax 70 Gy (<5%)	
Brachial plexus	PRIM	0.1 cc	Dmax 60 Gy	Dmax 66 Gy	SEC in selected
Thyroid Gland	SEC	V45	<50%		
Submandibular gl	SEC	Dmean	<35 Gy		
Constrictor pharyngeal mm	SEC	Dmean	<50 Gy		
Lacrimal gland	PRIM	Dmean	26 Gy		SEC in selected cases
Lens	PRIM	Dmax	<4 Gy	<6 Gy	SEC in selected cases
Retina	PRIM	0.1 cc	Dmax 54 Gy	Dmax 60 Gy	
Pituitary gland	SEC	Dmax	<50 Gy		
TM joints	PRIM	0.1 cc	<70Gy		

Contouring indications and structured atlases [60,61]can be herein found:

- http://groups.eortc.be/radio/res/gregoiratlas/ln_levels_neck.pdf;
- http://www.rtog.org/LinkClick.aspx?fileticket = TjrmNiHXly8%3d&tabid = 229.

Dose and constraints reporting and recording should be standardized according to Institution guidelines.

The tolerance parameters reported here have only an indicative value since they may vary significantly among individual patients based on genetic factors, co-morbidities, concurrent or previous treatments, clinical status, habits and supportive treatments. Moreover, it should be noted that dose fractionation may influence the risk of both acute (weekly total dose) and late (d./f.) reactions. The doses reported below should to be considered equivalent to 2 Gy d./f.; their use at a lower d./f. would be conservative, or allow for extra safety.

### PTV dose (volume) objectives

The process of developing a treatment plan consists of 3 components: (1) the definition and description of the “planning aims” and the desired absorbed-dose levels; (2) a complex beam delivery “optimization” process to achieve and, if needed, modify the initial “planning aims”; (3) a complete set of finally accepted values, which becomes the “prescription”. When optimized, the absorbed-dose distribution is accepted by the physician.

In the Literature different parameters regarding PTV prescription and dose homogeneity are reported [62-64].

Hereinafter, the prescription dose is defined as the isodose encompassing at least 95% of the PTV.

Target dose restrictions include the followings: no more than 20% of any PTV could receive >110% of its prescribed dose, no more than 1% of any PTV would receive <93% of the prescribed dose, and no more than 1% or 1 cc of the tissue outside the PTV would receive >110% of the dose prescribed to the primary target [17].

### Planning issues

- *Split Field vs. Whole Field IMRT.* Most Institutions would plan IMRT for HN cancers considering the whole volume (‘whole field IMRT’, WF-IMRT). However, some Authors, especially in absence of positive lymph nodes and for tumours above the arytenoids (i.e. tonsil) propose the use of IMRT for the upper part of the volume keeping a low, anterior-posterior parallel-opposed beam approach to cover the lower neck and the supraclavicular nodes (‘split field IMRT’, SF-IMRT). The issue has been highly debated in the literature [62,65]. Although there are pro and cons for each strategy, our preference is to use WF-IMRT especially when nodes are present in the mid/low neck to avoid underdosing of the volume junction.
- *Static IMRT vs. VMAT*. IMRT takes several minutes to be delivered and this may be uncomfortable for the patient and workload intensive for the Institution (a typical time slot for treatment reaches 30 minutes including IGRT). Tomotherapy allows for the delivery of volumetric IMRT in a shorter period of time and sharper dose distributions with doubtful clinical impact [66]. Linac-based volumetric IMRT, VMAT or Rapid Arc, offers the advantage of less monitor units and shorter delivery times over static IMRT [67], but no clear advantage (and possibly disadvantage) in terms of target coverage and OAR sparing. Although this is a rapidly evolving field, the impression is that VMAT/RA may not offer the same dose distribution of IMRT/Tomotherapy for complicated cases [67]. Within this uncertainty, it seems reasonable to implement VMAT/RA, at least initially, in simple cases and with a dosimetric comparison to standard IMRT.
- *Tissue deformation during treatment*. Most patients undergoing RT ± CT for HNC may lose a significant proportion of their weight (on averages 6% to 12% of pre-treatment body weight [68]) during a 7-week course. Sometimes, large nodal masses show a dramatic shrinkage [69]; especially when adjacent to surrounding normal structures (cord, parotids), the dose distribution during treatment may deviate significantly during treatment compared to initial planning [70]. Finally, it has been shown that almost all OARs undergo significant changes during treatment, with about 30% of shrinkage for major salivary glands, 10% for muscles (with the exception of constrictors) and 15% increase for larynx and constrictors [71]. The cord, the brain and other nervous tissue structures seem unaffected. An adequate supportive/nutritional treatment (including enteral feeding) has proven to limit weight loss during treatment, increase patient compliance and ultimately patient’s quality of life [72]. Which is the role of daily IGRT and adaptive re-planning in this setting is still undefined. Daily IGRT may reduce both systematic and random errors and thus may be preferable over weekly KV orthogonal films that address only the former [73], though no firm clinical data support this recommendation. One issue with the HN district is the relative motion of structures, i.e. the mandible and shoulders relatively to the neck and the deformability of structures (i.e. neck curvature). These limit the success of corrections using translations. Re-planning should be currently limited to cases where the changes in anatomy jeopardize the clinical outcome, i.e. increase in dose to the cord due to shrinking neck masses [70]. Fortunately, only a minority of cases (<5%) show such modifications during treatment, and overall most patients still fail within the high dose region suggesting that geographic misses both at planning and during treatment are not a prevalent cause of failure. At present, rescanning of the patient during treatment (with subsequent recalculation of the dose distribution on the new planning CT ± re-optimization) is considered at some Institutions mainly in patients with significant (>10%) weight loss or (nodal) tumour changes.
- *Dental artefacts and tissue heterogeneity calculation*. Metal artefacts, in form of fixed intracavitary dental alloys, may induce dose alterations with dose enhancement that can lead to adverse tissue complications in OARs in contact to or near PTVs, and dose attenuation that may potentially spare cells within the target volume.
- Two methods have been proposed to limit tissue heterogeneity. A method that uses a model with four different material classes: air, soft tissue, bone, and metal. Pixels belonging to the same class are assigned to the same representative Hounsfield unit (HU). The HU of the pixels in the streaks resulting from the presence of metal are replaced as much as possible by the Hounsfield units of the soft tissue class or bone class [74], thus reducing artefact influence on dose calculation. Another method uses MV-CT imaging obtained with the treatment machine, with the patient in the treatment position, that can be registered with the simulation kV-CT scan for the purposes of structure delineation, dose calculation, and treatment planning [75].
- Alternatively, a simple/basic empirical method is to override tissue density in the metal region after contouring. In this setting it is always prudent to rerun the dose calculation with the original tissue heterogeneity to better appreciate significant deviations.
- *PTV and skin*. Whenever the tumour or clinical target volume approaches the surface of the skin, part of the PTV can extend into the build-up region of incident photon beams or even into the surrounding air (so-called in-air PTV). Most dose-computation algorithms cannot accurately compute absorbed dose in build-up regions. This will lead to a convergence error, i.e., the optimizer does not reach a global minimum for the objective function [76].
- Two solution are usually proposed: i. The PTVs are cropped so that they are restricted to 3 mm below the contoured body surface to prevent optimization issues in the build-up region except for areas where the skin is considered a part of the CTV. In the latter situation, a bolus is suggested, as stated in the “simulation” chapter. In addition, the PTV receiving lower dose is cropped from the overlapping higher dose PTV to facilitate optimization. After optimization, a skin flash region (virtual bolus) is used to obtain a sufficient fluence in surrounding air to cover the original PTV. ii. Planning-target-volumes are subdivided and a relaxation of the planning aims is used (ICRU 83).
- *PTV overlapping OARs.* The second solution proposed in previous situation is the preferred one when there is a PTV/PRV overlapping knowing that it is a trade-off between the coverage of the PTV with the aimed dose and the saving of OARs within the constraints.

### Oral cavity

Cancers of the oral cavity are usually managed by upfront surgery and postoperative (chemo)RT. RT can be used as primary therapy for small (T1, T2) tumours of the oral cavity. Best results are with a combination of external beam radiation and brachytherapy.

IMRT is often used for oral cavity SCC to limit the dose to OARs and primarily to the parotid glands. However, compared to 3DCRT, it is associated with longer delivery or treatment session time. This may not be an ideal option for patients, who, especially after extensive surgeries, due to accumulation of secretions in the mouth, may not tolerate the supine position. Sometimes suctioning secretions before treatment delivery helps to maintain the treatment position during IMRT. Furthermore some initial reports with IMRT register marginal failure especially in patients with perineural invasion raising the question whether conventional opposed lateral fields might be a better option, also highlighting the importance of target delineation [82] [83].

### Dose/fractionation remarks

Table 3 shows the commonly used fractionation regimens in the curative setting.

**Table 3:** **Suggested fractionation regimens for definitive treatment of oral cavity cancers**

**Author**		**D (Gy)**	**d (Gy)**	**Fxs**	**OTT (wks)**	**Comment**
Daly et al., [83]	CTV1	66	2.2	30	6	With concurrent CT [83]
Yao et al., [82]		70	2	35	7	Sequential boost [82]
	CTV2	54	1.8	30	6	
Yao et al., [82]	CTV3	54	1.8	30	6	[82]
Daly et al., [83]		50.1	1.67	30	6	[83]

Table 4 shows the commonly used fractionation regimens in the post-operatve setting:

**Table 4:** **Suggested fractionation regimens for postoperative setting of oral cavity cancers**

**Authors**		**D (Gy)**	**d (Gy)**	**fxs**	**OTT (wks)**	**Comment**
Daly et al., [83]	CTV2	66	2.2	30	6	For positive margins or ECE
Yao et al., [82]		64-66	2	32-33	6.5	Sequential boost for extracapsular extension, positive or close margins, bone or soft-tissue involvement.
		60	2	30	6	
		63	1.8	30	6	
	CTV3	54	1.8	30	6	
Daly et al., [83]		50.1	1.67	30	6	
		58.1	1.66	35	7	

A surgery-to-RT interval of <6 weeks improves local-regional control.

### Primary-tumour contour

#### Definitive treatment

1. Table 5 describes anatomical landmarks in contouring various oral subsites

**Table 5:** **Anatomical landmarks in contouring various oral subsites**

**Sub-site**	**Cranial**	**Caudal**	**Anterior**	**Posterior**	**Lateral**	**Medial**
Oral tongue/ Floor of the Mouth (FoM)	Superior aspect tongue	Hyoid bone	Symphysis menti	Anterior oropharyngeal mucosa	To mandible. Includes ipsilateral parapharyngeal space	Ipsilateral tongue/FoM in well lateralized tumours. Contralateral mandible in midline or advanced tumours
Buccal mucosa	Inferior aspect zygomatic arch/hard palate	Hyoid bone	Angle of mouth	Oropharyngeal mucosa.	To overlying skin	Oropharyngeal mucosa. Contralateral parapharyngeal space spared
				Infratemporal fossa should be included in HNCPs with involvement or proximity to inferior alveolar nerve		
Retromolar Trigone	Superior aspect soft palate/hard palate	Hyoid bone	Junction of posterior third and anterior two thirds of the tongue	Oropharyngealmucosa	To mandible.	Oropharyngeal mucosa
						Contralateral parapharyngeal space spared
					Includes ipsilateral parapharyngeal space	
Hard palate	Superior aspect of hard palate +10 mm	Hyoid bone	10-15 mm anterior margin on GTV into palate	Anterior aspect oropharyngeal mucosa	To mandible / medial pterygoid muscle on both sides. Includes both Parapharyngeal spaces.	To mandible/medial pterygoid muscle on both sides.
						Includes both parapharyngeal spaces.
2. At least a 1 cm margin around the GTV should always be added. In cases where the extent of the tumour is difficult to visualise it’s preferable to use a 15 mm margins. CTV to PTV expansion also need to consider the local motility of the target (i.e. oral tongue) with respect also to the use of immobilization devices (i.e. tongue depressor) and their reproducibility. Anisotropic expansions (up to 10 mm) are often used to accommodate regions at higher motility (i.e. the oral tongue).

### Postoperative treatments

A positive margin on the oral tongue can be an indication for re-resection. Even when converted to negative, an *initial positive margin* carries a worse prognosis than an initially negative one [84] and thus should be considered at high risk of failure. In oral tongue cancers, *perineural invasion* (PNI) is an adverse feature that qualifies for adjuvant treatment at the primary site; moreover, in case of PNI, the value of a negative margin is somewhat questionable due to the possibility of a skip metastasis along the nerve route. In case of direct nerve invasion, e.g. if the inferior alveolar nerve is positive, the *infratemporal fossa* should be included in the radiation field because cancer can spread retrograde through the nerve into infratemporal fossa and to the skull base. The *infratemporal fossa* should also be included in the radiation field for tumour adjacent to this nerve with extensive perineural invasion and for tumour invading the pterygoid muscle, which is most commonly seen in patients with retromolar trigone cancers [82].

The CTV3 includes the whole operative bed and reconstruction site (i.e. free flap, mandibular reconstruction).

### Lymph-node station contour

**Table 6:** **Guidelines for contouring neck levels** [57]

	**cNo**	**Ipsilateral N+**	**Comment**
Tongue	Bilateral I-IV	V if N2-3	Excluding IIb
Floor of mouth (well lateralized)	Bilateral I-III	IV and V if N2-3	Excluding IIb
Hard palate	Bilateral Ib, IIa, III, RP	Add bilateral Ia, IV, V if N2-3	Excluding IIb
Upper retromolar trigone	Bilateral Ib, IIa, III, RP, ipsilateral Ia	Add contralateral Ia, bilateral IV and V	Excluding IIb
Lower retromolar trigone	Ipsilateral I, II, III	Add ipsilateral IV, V	Excluding IIb
Buccal mucosa	Ipsilateral Ib, IIa, III	Add ipsilateral Ia, bilateral IV and V	Excluding IIb

The neck (at least on the side of the disease) is often dissected during the removal of the primary tumour. As such, it is usually included in the lowest dose level regardless of pathology findings (just because part of the operative bed and thus potentially contaminated). A further dose is best judged based on the final pathology findings (high risk features, see above). Sometimes the clinically negative neck is not dissected (usually when the indication for RT is based on other findings that are already known at the time of surgery-i.e. contralateral to cN+). When dissected, indications for adjuvant treatment include: pN > 1 (multiple pathologically involved nodes/multiple levels); single node with extracapsular extension; incomplete neck dissection (i.e. lack of level IV dissection in patients with pathologically positive level III); atypical node location (i.e. skip metastasis in level III) (see Table 1) for dose level definitions).

Treatment of the neck can be unilateral for well lateralized lesions (i.e. buccal mucosa), in absence of cN2-N3 ipsilateral neck nodes [85] (see Table 6).

*Oral tongue primaries carry a higher risk of contralateral nodal disease* than other oral cavity sites because of the rich bilateral lymphatic drainage [86], therefore the bilateral neck should be irradiated in all oral tongue cancer patients with involved ipsilateral nodes, especially in high grade tumours or advanced T stages [82].

1. Oral tongue drainage is to ipsilateral level Ib and level II, III and even level IV with an incidence of skip metastasis of 10-15% [87]. An adequate elective neck dissection for oral tongue SCC includes levels Ib-IV. Five to ten percent of oral tongue cancers have bilateral lymph node metastases [88,89].
2. For oral cavity SCC, the risk of positive nodes is strictly correlated with the depth of primary tumour invasion. Three to 9 mm tumours have a 44% risk of positive nodes and a 7% risk of local recurrence; >9 mm thickness is associated with a 53% risk of subclinical nodes and 24% of local recurrence [90,91].
3. The floor of mouth and tip of the tongue drain to levels *Ia*, Ib and II. The incidence of bilateral lymph node involvement is relatively high because many lesions are near or cross the midline [88,89].
4. Tumours of the buccal mucosa and hard palate not exceeding midline drain to ipsilateral level Ib.
5. For well-lateralized lesions (at least 1.5 cm from midline) only the ipsilateral neck should be considered for treatment, especially in absence of N2-N3 disease (except for oral tongue, see above).
6. In general, for cN0-1, elective nodal stations include Ib-III with the exceptions of IIb (Table 6). The addition of level Ia is for lesions of the floor of mouth, oral tongue and lower gingiva. Adding level IV is for lesions of the oral tongue, especially if in the posterior 2/3.
7. The likelyhood that an oral cavity cancer involving cervical lymph nodes of levels I to III would also involve level IV is generally stated as 7% to 17%, and the corresponding rate for level V is 0 to 6% [92,93];
8. For cN2-3, elective irradiation of level V is advised; in case of extension to the tonsillar region, also retropharyngeal nodes should be included.

### Nasopharynx (NPX)

Standard treatment of nasopharyngeal carcinoma is definitive RT ± CT.

*Target definition remarks (Table**7**)*

**Table 7:** Standard anatomic limits of Nasopharynx

ᅟ	ᅟ
Anterior	Posterior fourth to third of the nasal cavity and maxillary sinuses (to ensure pterygopalatine fossae coverage)
Posterior	Anterior third of clivus (entire clivus if macroscopic infiltration), retrostyloid space
Lateral	Lateral parts styloid processes (parapharyngeal space)
Cranial	a. Skull base (foramen ovale and rotundum bilaterally must be included for all cases),
	b. Inferior half sphenoid sinus – anterior half clivus (entire clivus and top sphenoid sinus if macroscopic infiltration or in T4 cases). For lesions confined to the nasopharynx, the pituitary fossa can be excluded from the irradiated volume [97].
	c. The cavernous sinus should be included in high risk patients (T3, T4, bulky disease involving the roof of the nasopharynx)
Caudal	Soft palate

1. CTV1 = GTV+ a margin of ≥ 5 mm should be given circumferentially but can be 0–1 mm if anatomical barriers are present. CT and MRI should be used to identify the primary tumour location [20,94] (Figure 2). Indeed, the clivus and nerves are best seen on MRI.
2. CTV 2 = whole NPX (Table 7) should be included in high risk volume regardless T site [20,95,96]. Indeed Sham observed that only 7% of patients had involvement of one subsite regardless of T site [95]. The most outer boundary of CTV2 should be at least 10 mm from the GTV.

*Pathology remarks*

Histological subtypes: the old WHO classification of nasopharyngeal carcinomas [98], yet widely used in literature, should be replaced by the new one [99] in order to avoid confounding factors:

- WHO type 1 = keratinizing carcinoma
- WHO type 2.1 = non keratinizing differentiated carcinoma
- WHO type 2.2 = non keratinizing undifferentiated carcinoma (with lymphoepitelioma variants)
- WHO type 3 = basaloid SCC (a rarity)

While subsite only is not a predictor of treatment outcome, histological subtypes are strongly variables by race and strongly influence prognosis:

- The asian population have a greater proportion of non keratinizing tumours, up to 90% [100], while white Caucasian race has a 60-70% rate of non keratinizing tumours [101,102]. WHO Type I, II differentiated, and II undifferentiated lesions have 41%, 56.1% and 68.5% 5-year survival rates, respectively [103].
- Moreover WHO subtypes influence the risk of nodal and distant metastases: Type 1 and 2 have a 60% and 90% rate of nodal involvement respectively at diagnosis; Type 1 and 2 have 5-8% and 30-40% distant metastases rate respectively at diagnosis [104].
- Of note, the same radiation dose is prescribed for all variants.

*Dose/fractionation remarks*

Table 8 shows the commonly used fractionation regimens:

**Table 8:** **Suggested fractionation regimens for NPX**

**Author**		**D (Gy)**	**d (Gy)**	**fxs**	**OTT (wks)**	**Comment**
	**CTV1**	**70**	**2**	**35**	**7**	
N. Lee et al., 2009		69.96	2.12	33	6.5	RTOG0225 [20]
K. Kim et al., 2009		67.5	2.25	30	6	[105]
Peponi et al., 2010		66-69.63	2.2-2.11	30-33		[106]
	CTV2	63	1.8	35	7	
N. Lee et al., 2009		59.4	1.8	33	6.5	RTOG0225 [20]
K. Kim et al., 2009		54-60	1.8-2	30	6	[105]
	CTV3	58.1	1.66	35	7	
		56.1	1.7	33	6.5	
N. Lee et al., 2009		50.4	1.8	28		RTOG0225 [20]
K. Kim et al., 2009		48	1.6	30	6	[105]
Peponi et al., 2010		51	1.7	30		[106]

*Primary-tumour contour*

- Anatomic considerations (see also Table 7).

1. NPX is usually divided into 3 subsites: a) postero-superior wall: extends from the level of the junction of hard and soft palates to the base of skull; b) lateral wall: including the Rosenmüller fossa; c) inferior wall: consists of the superior surface of the soft palate.
2. The *pharyngobasilar fascia* is a robust fibrous aponeurosis situated between the mucous and muscular layers and covers the pharyngeal constrictor muscles; it is bound inferiorly by the superior pharyngeal constrictor, superiorly by the base skull, and anteriorly by the posterior border of the medial pterygoid laminae; it serves to attach the superior pharyngeal constrictor muscle to the base of the skull at both the basal part of the occipital bone and the petrous portion of the temporal bone; the Eustachian tubae generate an escape route for the tumour that frequently exceeds this aponeurosis and reaches the parapharyngeal and masticatory space.
  Neoplastic-behaviour considerations
3. Nasopharyngeal neoplasms typically arise near the Rosenmuller fossa.
4. The natural local growth follow four directions [107,108] and this should be taken into account for CTV contouring:
  - **Anterior**:
    i. nasal cavity;
    ii. masticatory space via pterigomaxillary fissure;
    iii. carotid canal via anterior foramen lacerous;
    iv. intracranial (medial cranial fossa) via foramen rotundum;
    v. orbital cavity and cranial anterior fossa via superior orbitae fissure.
  - **Lateral:**
    i. masticatory space via Eustachian tubae,
    ii. posterior cranial fossa via giugular foramen and giugular internal vein;
    iii. cavernous sinus via mandibular nerve and foramen ovale.
  - **Posterior** : prevertebral muscles and vertebrae via prevertebral fascia;
  - **Inferior**: oropharynx

- Therapeutic considerations

1. Induction CT is sometimes used for neoadjuvant purposes to shrink and downsize the tumour especially when it is abutting OARs whose tolerance is inferior to the prescribed GTV dose (i.e. brainstem). In this case, the pre-induction target should receive full dose [29,109] (Figure 2), but the dose to the original site of disease is often limited to the tolerance of surrounding OAR (i.e. 60 Gy, optic pathways) while the residual tumour is prescribed/delivered a full dose.

The patient should be simulated supine with the head hyperextended [110] to provide adequate separation between the primary lesion/retropharyngeal lymph node and the upper neck field and to avert eyes from the primary volume, or in neutral position.

*Lymphnode-level contour*

- Anatomic considerations.
  There are three distinct pathways of lymphatic drainage from the nasopharynx: i. *Postero-inferiorly* to the retropharyngeal nodes (including the node of Rouviere); ii. *Directly* to superior deep cervical nodes; iii. *Laterally* to mastoid and spinal accessory nodes (level V).
  The initial lymphatics (including capillary network and precollectors) arise in the wall of the nasal fossae and the nasopharynx.

The lymphatic collectors run into the parapharyngeal space to the lateral pharyngeal and retropharyngeal lymph nodes. They run through the lateral wall of the nasopharynx and form two lymph collectors (lateral and medial) that descend laterally to the pharyngeal wall in the parapharyngeal fat tissue:

i. *the lateral one* is situated on the lateral side of the external carotid artery;
ii. the medial is located one medially along the external carotid artery.

The lateral group provides lymphatic drainage from the lateral walls (including Rosenmuller Fossa) directly into deep nodes of the posterior triangle (upper level V) while the medial trunk drains to the superior deep cervical nodes (levels II and III) [111,112].

- Neoplastic-behaviour considerations
  1. The primary lymphatic drainage of nasopharyngeal carcinoma is to retropharyngeal, II and Va nodal levels. Setting the cranial border of level IIb node at the skull base should be considered when delineating nodal target volume [113].
  2. The secondary lymphatic drainage is to levels III and Vb [114].
  3. Level IV is involved in 10% of the cases [115].
  4. Level Ib is rarely involved; it has to be included only in case of clinical adenopathy evidence [115]. It should be noted, however, that the posterior part of level Ib was routinely irradiated in the old days [59].
  5. Lymphatic drainage is bilateral. Therefore, both sides of the neck are at risk [41] (Table 9).

**Table 9:** **Guidelines for contouring bilateral neck levels (negative on imaging)**

**Side**	**Level**	**Risk**	**Remarks**
Bilateral	Retropharyngeal, II, III, Va	High	
	IV, Vb	Low	Higher risk when level III is clinically involved
	IB	Very Low	Omit or include only in case of neck node positivity
  6. Retropharyngeal nodes (top of C1 to bottom of C2, sometimes C3 [108,116]) should be always included in the CTV2.

Few studies have investigated the option to withhold elective treatment for cN0 patients. One of them found a high regional salvage rate with either surgery or RT [117]. It should be noted, however, that patients with residual persistent disease in the neck or who fail in the neck have a higher risk of distant metastases than patients who do not fail in the neck. Therefore, comprehensive neck irradiation (including levels II-V) is always advocated for NP.

### Oropharynx

The oropharynx is usually divided into 4 sub-sites: tonsil, base of tongue, pharyngeal walls and soft palate. While sub-site only is not a predictor of treatment outcome, HPV related tumours, with a more favourable prognosis compared to their alcohol and tobacco counterparts, usually originate from the base of tongue or tonsils. *Currently there are no practical implications or differences between HPV positive and negative tumours.*

*Dose/fractionation remarks*

Table 10 shows the common fractionation regimen.

**Table 10:** **Suggested fractionation regimen for oropharyngeal cancer**

**Authors**		**D (Gy)**	**d (Gy)**	**Fxs**	**OTT (wks)**	**Comment**
	**CTV1**	**70**	**2**	**35**	**7**	
Eisbruch [17]		66	2.2	30	6	T1-2 in absence of conc chemo (RTOG 00–22)
Sanghera [118]		55	2.75	20	4.5	Retrospective analysis
	CTV2	63	1.8	30	6	
Eisbruch [17]		60	2	30	6	T1-2 in absence of conc chemo (RTOG 00–22)
	CTV3	58.1	1.66	35	7	
Eisbruch [17]		54	1.8	30	6	T1-2 in absence of conc chemo (RTOG 00–22)
Sanghera [118]		41.25	2.75	15	3	

*Primary-tumour contour*

**Figure 3:**
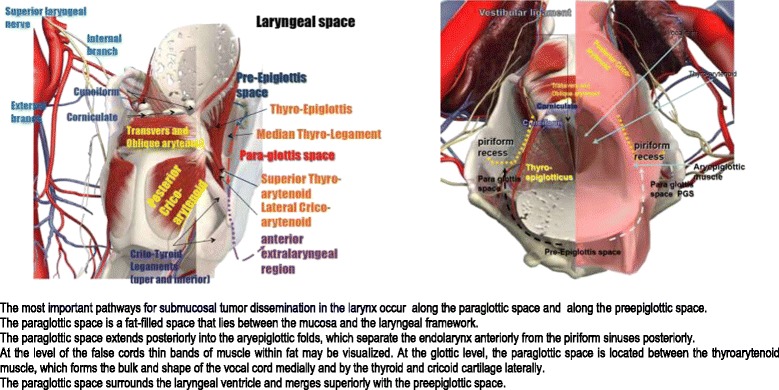
**Laryngeal anatomy.**

- Historical perspective:

1. In the standard 3-field era, the whole oropharynx was included in the low dose region and the primary tumour boosted to the final dose. Whether the whole oropharynx should be included in the low risk region is somewhat controversial and no definitive recommendations can be made. However, some Authors, especially in presence of HPV related tumours (that seem to have a lower risk of field cancerization) tend to limit the low risk volume (CTV3) to the sub-site involved or, at most, the adjacent one (i.e. in case of a tonsillar tumour, CTV3 would include the involved tonsil and the tongue base but not the contralateral tonsil).
2. In the old days, a typical initial field for a tonsillar tumour included the whole pterygoid plates up to the base of skull; therefore it is recommended that CTV3 cover the whole pterygoids as well.

- Clinical and anatomical considerations

1. Trismus is a surrogate of medial pterygoid muscle invasion and in this case a generous margin along with the medial pterygoid muscle should be contoured during CTV1 definition;
2. The anterior extent of the GTV in the tongue base is best evaluated via contrasted CT or even better with MRI; due to subclinical anterior extension, especially for infiltrative primary tongue base tumours, a generous margin (1.5-2 cm) around both CTV1 and CTV3 should be added anteriorly;
3. Patients with dentures that are likely to create visual artefacts should be positioned with the head in a different position (more flexed or extended); moreover, the location of the primary tumour should be driven by a different study, i.e. MRI;
4. After primary tumour (surgical) removal, unless the original location of the primary is clearly available and visible on a dedicated study, the whole sub-site should be part of CTV;
5. Parapharyngeal space is a locus *minoris resistentiae* and needs to be included in the CTV in the case of possible/suspected local extension and/or retro/parapharyngeal node involvement (Figure 3).

*Lymphnode-level contour*

1. The primary lymphatic drainage of oropharyngeal SCC is to levels II, III and retropharyngeal nodes. Bilateral spread is fairly common for lesions that approach midline (such as in the case of the tongue base and soft palate), while it is relatively rare for well lateralized lesions (at least 1.5 cm from midline as in the case of tonsil primaries) in absence of multiple or large (>3 cm) lymph nodes; in addition, lesions arising or involving the soft palate have an increased risk of level I (Ib) involvement;
2. When the primary tumour is well lateralized (e.g. tonsil) *and* the ipsilateral neck shows limited involvement (N0-1, questionable N2a), contralateral neck coverage can be omitted;
3. Some Authors [59,119] would limit level IB coverage to its most posterior part. The anterior extent of the contour of level IB when included in CTV3 would stop at the anterior extent of the submandibular gland and, therefore, exclude the triangular fat space lateral to the deep extrinsic muscles of the tongue.
4. Retropharyngeal nodes (top of C1 to bottom of C2, sometimes C3) should be always included in the CTV3 unless suspicious (>5 mm).
5. In case of suspicious neck nodes (as previously defined) and lymph node enlargement in other sites than ipsilateral levels II and III, the suspicious finding and not the whole level should be contoured as CTV2.
6. Table 11 provides some indications for contouring ipsilateral and contralateral neck levels that are negative at imaging.

**Table 11:** **guidelines for contouring ipsilateral and contralateral neck levels negative at imaging**

**Side**	**Level**	**Risk**		**Remarks**
Ipsilateral	Ib	Low	CTV3	Higher risk for soft palate/oral cavity involvement or multiple positive neck levels-CTV2
	II-III	High	CTV2	Lower risk when the whole neck is negative (cN0)-CTV3
	IV	Low	CTV3	Higher risk when level III is clinically involved- CTV2
	V	Very low	CTV3 or omit	
Contralateral	IB	Very low	Omit	Higher if the contralateral II-III lymph node level is clinically positive or the soft palate is involved
	II-III	Low	CTV3	Higher risk if the contralateral level IB is clinically positive
	IV	Low	CTV3	Higher risk when level III is clinically involved
	V	Very low	Omit	Omit unless suspicious nodes are found

**Table 12:** **Suggested fractionation regimens for hypo-pharyngeal and laryngeal cancer**

**Author**		**D (Gy)**	**d (Gy)**	**Fxs**	**OTT (wks)**	**Comment**
	**CTV1**	**70**	**2**	**35**	**7**	
Lee et al. [122]		70	2.0	35	7	2-years PEG dependent: 15%
Studer et al. [121]		69.6	2.11	33	6.5	
Miah et al. [123]		63	2.25	28	5.6	Arm 1 (DL1)
		67.2	2.4	28	5.6	Arm 2 (DL2)
	CTV2	63	1.8	35	7	
Lee et al. [122]		59.5	1.7	35	7	
	CTV3	58.1	1.66	35	7	
Lee et al. [122]		56.0	1.6	35	6.5	
Studer et al. [121]		54	1.64	33	6.5	
Miah et al. [123]		51.8	1.85	28	5.5	Acute Grade 3 (G3) dysphagia was higher in DL2 (87% DL2 vs. 59% DL1)
		56	2	28	5.5

### Hypopharynx

SCC of the hypopharynx is usually diagnosed at a later/advanced stage. Moreover, due to local (submucosal) invasion, it is usually considered more aggressive/less radioresponsive than its laryngeal counterpart. Concomitant CT is usually indicated for T3-4 or anyT N+ [120-123], but some Authors in monoinstitutional experience [124,125] claim the use of concomitant CT also for earlier T stage (T2) lesions even if the only two large randomized trials specifically addressing the hypopharyngeal cancer subset [126,127] with few T2 patients included, used sequential or alternate ChT and RT and not concomitant treatments. In patient unfit for standard concomitant CT, induction CT or the concomitant association with Erbitux can be considered [11].

Due to concerns regarding treatment-related late toxicities, hypofracionation delivered with SIB is not recommended [122,128].

### *Target definition remarks*

CTV2. After surgery, CTV2 would include the whole laryngo-pharynx bed with particular attention to the cranial and caudal extent of resection (i.e. cervical oesophagus) and the site of dissected positive lymph nodes;

*Dose/fractionation remarks*

*Primary-tumour contour*

- Historical perspective:
- In the old days, the lower extent of tumour coverage (cricoid cartilage) needed a low neck junction that was particularly troublesome in patients with a short neck; nowadays with IMRT, the issue about adequate coverage of the caudal part of the CTV is overcome [128].
- Clinical and anatomical considerations
- The hypopharynx consists of the pyriform sinuses, postcricoid region and posterior pharyngeal wall. The anatomic boundaries of the hypopharynx include the ariepiglottic folds (superiorly), the cricoid cartilage (inferiorly), the prevertebral fascia (posteriorly) and the larynx (anteriorly).
- Unlike the larynx, the hypopharynx has no clear barriers to cancer extension. The boundary between the oropharynx and hypopharynx is theoretical and the boundaries between different subregions are more anatomical landmarks that real barriers.
- Because submucosal extensions are hardly visible, it is preferable to have larger volumes to reduce the risk of recurrence. Therefore, CTV2 would encompass 1–1.5 cm of normal mucosa cranial and caudal to CTV1 [129].
- The lower extent of the tumour is best evaluated with a dedicated esophagoscopy.
- Especially for lesions involving the medial wall of the pyriform sinus, the posterior part of the larynx (arytenoids and interarytenoid region) should be part of the CTV1 as well.
- Involvement and violation of the prevertebral fascia can be appreciated clinically (tumour fixation) and on imaging (MRI); in case of prevertebral space invasion, the lesion becomes unresectable and the margins for cranio-caudal microscopic tumor spread would be wider than usual.
- Indications to boost the stomal region after major surgery basically duplicate those for laryngeal primaries.

*Lymphnode-level contour*

1. Tumours of the hypopharynx have a high tendency to spread to lymph nodes.
2. The risk of subclinical disease in lymph nodes is not strictly correlated to the stage of the primary tumour.
3. Stations at risk are reported in Table 13 [57,130].

**Table 13:** **Guidelines for contouring neck levels when negative at imaging in hypopharyngeal cancer**

**Site**	**N stage**	**Levels to be included**	**Remarks**
Pyriform sinus	N0	IIa-IV bilateral	V and RP bilateral, VI ipsilateral if oesophageal extension
Pyriform sinus	N+	IIa-V-RP bilateral, VI ipsilateral	Ipsilateral retrostyloid lymph nodes need to be included if clinical or radiological level II involvement is present.
Pharyngeal wall	N0	IIa-IV-RP bilateral, VI ipsilateral	V bilateral if esophageal extention
Pharyngeal wall	N+	I-V, RP bilateral, VI ipsilateral	Ipsilateral retrostyloid lymph nodes need to be included if clinical or radiological level II involvement is present.
4. Lymph node level VI has to be encompassed if oesophageal extension is present.
5. Ipsilateral retrostyloid lymph nodes should be included if clinical or radiological level II involvement is present.

### Larynx

*Dose/fractionation remarks*

Table 12 reports the most used fractionation schemes for laryngeal cancer. The standard dose for T1N0 of the glottic larynx is 66 Gy /33 fxs/6.5 weeks. For T1 glottic lesions, the d./f. used within 3DCRT has been up to 3.43 Gy [131] (Total dose 55Gy/16 fractions); there is limited experience with IMRT for this fractionation, and thus the recommendation is to limit the d./f. to 2.25 Gy to 56.25–63 Gy [132] in the context of IMRT. For T2 glottic lesions, RTOG 9512 found only trend in favour of hyperfractionation over standard fractionation [133].

For more advanced lesions (T3-4) of the larynx, there are consolidated data on the benefit of altered fractionation in terms of both local control and survival; the best outcome is reached with hyperfractionation [134]. However, hyperfractionation hardly fits into an IMRT technique for reasons that have been previously discussed; moreover with the advent of ChT, RT alone is rarely used for T3-4 lesions.

In the postoperative setting, the most used schedule is: 66 Gy in 33 fractions (2 Gy daily) to CTV1, 59.4 Gy in 33 fractions (1.8 Gy daily) to CTV2 (optional) and 54,12/56,1 in 33 fractions (1.64-1.7 Gy daily) to CTV3.

After subtotal laryngectomies (horizontal supraglottic laryngectomy-HSL, near total laryngectomy, hemilaryngectomy) there is the concern that adjuvant RT (±ChT) may jeopardize the functional outcome of the residual or surgically spared larynx [135]. For example, after HSL (horizontal supraglottic laryngectomy), it has been found that 60 Gy radiation significantly increases the risk of laryngeal edema (over 50 Gy) [136]. Therefore, unless specifically indicated (positive resection margins) and after careful discussion with the patient, the dose to the larynx should be kept within 50 Gy (see also below) [137,138].

### Primary-tumour contour

The larynx is divided into three anatomic regions: Supraglottis — suprahyoid epiglottis, infrahyoid epiglottis, aryepiglottic folds (laryngeal aspect), arytenoids, and ventricular bands (false cords). Glottis — true vocal cords, including anterior and posterior commissures. Subglottis — subglottis, extending from lower boundary of the glottis to the lower margin of the cricoid cartilage [139].

Definitive treatments

- Anatomical considerations

1. Target volumes are defined according to the pathways of cancer extension in the larynx (Figure 3). Cancer of the larynx mainly spreads by direct extension (following the line of least resistance), prior to lymphatic spread and more rarely follows the vascular and neurological routes. The main way of diffusion of laryngeal cancer is to para-glottic and to the pre-epiglottic spaces through the anterior commissure, the thyro-epiglottic and thyro-arytenoid ligaments (upper and lower), the ligament and crico-thyroid membrane, the perforations of the sub-hyoid epiglottis, the bottom of the ventricle and laterally through the thyroid cartilage. These two spaces also play an important role for the T classification, as neoplastic invasion of these spaces upstages laryngeal cancer to T3.
  Therefore, if these structures are directly encompassed by GTV, CTV1 should include also pre-epiglottic and para-glottic space.

- Historical perspective

1. In the 3DCRT era the whole larynx or the majority of it was included in the CTV1; nowadays there is the tendency to include in the CTV1 only the expanded GTV; however, careful considerations about the depth of invasion, the natural history of disease, the presence/absence of anatomical barriers (i.e. continuity between pre-epiglottic and para-glottic spaces (Figure 3) and the quality of the imaging should always be taken into consideration; the use of anatomic compartments to define CTV boundaries is more adequate than the use of any arbitrary uniform expansion around the GTV, unless such boundaries are not well-defined, such as the anterior boundary in case of base of the tongue involvement [50]. Thus, the CTV1 corresponds to the GTV plus a margin of 5 mm to 1 cm or more, depending on the ways of diffusion or anatomical barriers (i.e. supraglottic tumours extending to the aryepiglottic folds will be outlined extending the superior margin of the CTV to 2 cm above the GTV; in case of infiltration of the base of tongue, a structure that does not present anatomical barriers, a margin of 1.5-2 cm is appropriate) and should not be modified according to a possible response to induction ChT.[140]. In correspondence of anatomical barriers GTV to CTV expansion should not exceed those structures (eg, prevertebral fascia, bone). In case of doubt of submucosal extension, it is better to enlarge the volumes to reduce the risk of recurrence.
2. The level of complexity required for the irradiation of laryngeal cancer is highly variable and IMRT may provide distinct advantages over 3DCRT in sparing OARs (parotids, especially when level IIb is part of the target, carotids for early stage lesions) and covering the target (avoiding field junctions and irradiating the subglottis especially in patients with a ‘short’ neck’). Though there are several dosimetric and even preliminary clinical experience with IMRT for T1-2 glottic cancers [141,142] or for locoregionally advanced glottic and supraglottic cancers [121-123], the clinical benefits of IMRT are usually speculative and the standard of care remains 3DCRT.

Table 14 provides guidelines for contouring primary tumor CTVs on planning imaging:

**Table 14:** **Guidelines for contouring larynx (according to subsites)**

**Supraglottic larynx**	**Superior limit**	**Inferior limit**	**Ipsilateral limit**	**Contralateral limit**	**Anterior limit**	**Posterior limit**
CTV 1	GTV + 0.5-1 cm margin	idem	Idem	idem	Idem	idem
CTV 2	Epiglottis, base of tongue (1 cm from CTV HD), arytenoids	Inferior border of chricoid cartilage	Hyoid bone, thyrohyoid, omohyoid and sternohyoid muscle laterally to thyroid cartilage, chricoid cartilage (plus pyriform sinus depending on GTV)	Hyoid bone, thyrohyoid, omohyoid and sternohyoid muscle laterally to thyroid cartilage, chricoid cartilage	Hyoid bone, thyroid cartilage, omohyoid and sternohyoid muscle anterior to pre-epiglottic space, thyroid cartilage, chricoid cartilage	Epiglottis, posterior limit of thyroid cartilage, arythenoids
CTV 3	Optional (add margin from CTV 2)	Idem	Idem	Idem	Idem	idem
Glottic larynx						
CTV1	GTV + 0.5-1 cm margin	idem	Idem	idem	idem	idem
CTV2	sub-hyoid epiglottis and pre-epiglottis space anterior to sub-hyoid epiglottis, hyoid bone, arytenoid	Inferior border of cricoid cartilage	Hyoid bone, thyrohyoid, omohyoid and sternohyoid muscle laterally to thyroid cartilage, cricoid cartilage (plus pyriform sinus and thyroid gland depending on GTV)	Hyoid bone, thyrohyoid, omohyoid and sternohyoid muscle laterally to thyroid cartilage, cricoid cartilage	Hyoid bone, thyroid cartilage, omohyoid and sternohyoid muscle anterior to pre-epiglottic space, thyroid cartilage, cricoid cartilage	Epiglottis, posterior limit of thyroid cartilage, arythenoids
CTV 3	Optional (add margin from CTV 2)	Idem	Idem	Idem	Idem	idem
Subglottic larynx						
CTV 1	GTV + 0.5-1 cm margin	idem	Idem	idem	idem	idem
CTV2	Epiglottis, base of tongue (1 cm from CTV HD), arytenoids	Superior limit of first tracheal cartilage (or lower depending on GTV)	Hyoid bone, thyrohyoid, omohyoid and sternohyoid muscle laterally to thyroid cartilage, chricoid cartilage, homolateral thyroid gland	Hyoid bone, thyrohyoid, omohyoid and sternohyoid muscle laterally to thyroid cartilage, chricoid cartilage	Hyoid bone, thyroid cartilage, omohyoid and sternohyoid muscle anterior to pre-epiglottic space, thyroid cartilage, chricoid cartilage	Retrochrycoid region, arythenoids, (upper oesophageal opening depending on GTV)
CTV 3	Optional (add margin from CTV 2)	Idem	Idem	Idem	Idem	idem

### Postoperative treatments

In the postoperative setting, features that are usually considered predictors of local relapse are: i. close or positive margins; ii. pT4; iii. extracapsular extension of the neck nodes. Other features (i.e. perineural spread) are less recognized and need to be considered on a case-to-case basis. In order to outline postoperative volumes, pre-surgery imaging studies and operative note(s) should be carefully reviewed. Moreover, the natural history of the disease plays also an important role.

In case of subglottic or soft tissue extension, the region of the stoma should be included in CTV2, while for upward lesions (i.e. lesions with significant anterior extension in the pre-epiglottic space) the base of tongue needs to be included in CTV2.

After subtotal laryngectomy, the decision to include the larynx within the low dose level (even in absence of high risk features), simply because it has been part of the operative bed, is controversial [135]. However, regardless whether the larynx is included or not, careful planning should avoid unnecessary overdosing of the larynx (>50 Gy) in absence of specific indication (e.g. if the decision to irradiate a removed level II node with extracapsular extension is made, the larynx should not receive a dose higher than 50 Gy even though the lymph-nodal area at risk needed a dose higher than 60 Gy). For high risk patients (R1 and pT4) CTV1 should include the whole remnant larynx with cranial and caudal extent of resection (e.g. subglottic extension) and the site of dissected positive lymph-nodes.

### *Lymphnode-level contour*

Anatomical considerations.

### Supraglottic larynx

The lymphatic drainage terminates in the ipsilateral level II nodes while a second component extends lateral and drains into nodes located at the junction of Levels II and III. There is occasionally a third component that drains into the nodes located in Level III nodes.

Tumours involving the supraglottic larynx are at risk of crossing lymphatic drainage. The risk of palpable lymph nodes at presentation is higher for epi-laryngeal tumours than for the rest of supraglottic one [143].

### Glottic larynx

The true vocal cords (TVC) form a natural barrier between the supraglottic and infraglottic larynx due to its paucity of lymphatic draining. At any rate, the lymphatic drainage of sovraglottic or subglottic larynx can be involved by advanced TVC carcinoma.

### Subglottic larynx

Lymphatics from the subglottic larynx drain to the mid and lower jugular lymph nodes (levels III and IV) and to the prelaryngeal (cricothyroid or Delphian) node. Subsequently, the pretracheal and paratracheal lymph nodes can be involved [144].

Tables 15 and 16 provide guidelines for contouring neck levels either in the case of negative or positive nodes on the imaging:

**Table 15:** **Guidelines for contouring neck levels (negative on the imaging)**

**Laryngeal Site**	**T stage**	**Levels to be included in CTV HR**	**Levels to be included in CTV LR**	**Remarks**
Supraglottic	T1-2	II-III (bil)	IV (bil),	
	T3-4	II-III (bil)	IV (bil), VI	
			VII only if subglottic extension	
			RP only if extension to pharynx	
Glottic	T1	None		
	T2	None/Questionable		Levels II and III if supragl ext Levels III and IV if subgl ext
		(see text)		
	T3-4	II-III (bil)	IV (bil), VI	
			VII only if subGL ext	
			RP only if ext to pharynx	
Subglottic	T1-2	III-IV (bil)		VII optional
	T3-4	II-IV (bil)	VI, VII, RP only if ext to pharynx

**Table 16:** **Guidelines for contouring neck levels (positive on imaging)**

**Laryngeal Site**	**TN stage**	**Levels to be included in CTV 2**	**Levels to be included in CTV 3**	**Remarks**
Supraglottic	T1-2- N+	II-III (bil)	IV bilat, V (on the side of positive neck)	
Glottis	T2 N+	II-III (bil)	IV bilat, V (on the side of positive neck)	
Subglottic	T1-2 N+	III-IV (bil)	V (on the side of positive neck),	
			VII	
Glottis/	T3-4 N+	II-IV (bil)	IIb if IIa positive, VI,	
Supraglottic/			V (on the side of positive neck),	
Subglottic			IB only on the side level II is pos*,	
			VII only if subGL ext	
			RP only if ext to pharynx	

Historical perspectives

1. For T2N0 of the glottis, the standard recommendation is to treat the larynx only, but the actual results are based on pre-IMRT cases. Conventional RT used to irradiate with two lateral opposing fields. Authors showed that portions of the neck lymphatic chain where inadvertently treated [59,133]. Therefore, it is advisable, in common practice, to irradiate levels II-III and III-IV especially when lesions extend into the supra or sub-glottis.
2. For primary lesions with subglottic involvement and clinically detectable lower neck nodes, the upper mediastinum (down to the level of the carina) was prophylactically covered with AP/PA fields. Therefore, inclusion of level VII in the CTV3 is advised in case of subglottic extension with significant and lower neck disease.
3. In the case of No, level IIb should not be irradiated.

Planning remarks

1. In case of suspicious neck nodes (as previously defined) it is unclear whether only the suspicious finding or the whole level should be included in the CTV2. For nodes belonging to lymph node stations different from ipsilateral levels II and III, the suspicious finding and not the whole level should be contoured as CTV2.
2. For lymph nodes that show gross extracapsular disease, a margin of 5[41]-10[53]mm around the visible GTV is usually added; in case of muscular infiltration by a pathological lymph node (i.e. sternocleidomastoid muscle), it is recommended to include at least the portion of the muscle surrounding the node [47]. Similarly, it would be appropriate to include the whole muscle (i.e. sternocleidomastoid muscle) in the lowest dose level when grossly infiltrated at some level.
3. After surgical removal of a lymph-node with extracapsular extension, the initial location of the node needs to be identified; careful planning should avoid underdosing of the surgical scar close to the pre-surgery location of the node (sometimes 3–5 mm skin bolus is necessary).
4. Subglottic cancer, transglottic cancer, and glottic cancer with subglottic extension have a higher risk of para-tracheal nodes involvement; for T > 2 even in N0 patients, this level should be included in CTV3 [57,145].
5. Ipsilateral Ib nodal level and parapharyngeal space extending to retrostyloid space should be included if level II nodes are positive.

### Paranasal sinuses and nasal cavity

*Target definition remarks*

RT may be indicated as postoperative (i.e. adenocarcinomas) or definitive treatment with or without concomitant CT. Whereas the definitive treatment leads to the irradiation of the usual 3 CTVs as described in the general part, the previous surgical approach (midfacial degloving, lateral rhinotomy, craniofacial, or endoscopic) may complicate the defintion of the volume boundaries. Detailed description of the surgical procedure and pathology report is mandatory. In the postoperative setting, there is no identifiable GTV and thus CTV1 is not available. CTV is represented by all areas at risk of containing residual microscopic disease and the choice between HR and LR depends on the risk for each sub-volume.

### *Dose/fractionation remarks*

Table 17 shows the commonly used fractionation regimens:

**Table 17:** **Suggested fractionation regimens for para-nasal sinus and nasal cavity**

**Definitive**		**D (Gy)**	**d (Gy)**	**fxs**	**OTT (wks)**	**Comment**
**Daly** **[** 146 **]**	**GTV**	**69.96**	**2.12**	**33**	**6.5**	
	CTV1	70	2	35	7	
Wiegner [147]		66	2.2	33	6.5	
	CTV2	63	1.8	35	7	
Daly [146]		59.4	1.8	33	6.5	
	CTV3	58.1	1.66	35	7	
Daly [146]		54.12	1.64	33	6.5	
Postoperative setting						
	CTV2	63	1.8	35	7	
Hoppe 2008 [148]		60-66	2-2.2	30-33	6	Especially for adenocarcinoma
	CTV3	58.1	1.66	35	7	
Hoppe [148]		54-54.12	1.8-1.64	30-33	6	

*Primary-tumour contour*

Table 18 describes the limits of the CTV2 for ethmoid and maxillary regions, according the suggestions of Stratt et al. [149].

**Table 18:** **Suggested CTV2 for ethmoid and maxillary sites**

	**Superior**	**Inferior**	**Lateral**	**Posterior**
Ethmoid	Cribriform plate should be included.	The inferior turbinate; In the case that the inferior border of the GTV allows a 10-mm margin around the original disease, the entire hard palate does not need to be included.	The nasal cavity, ethmoid sinuses, and the ipsilateral maxillary sinus and when indicated the volume should extend to the rectus muscle.	Include the sphenoid sinus. The retropharyngeal lymph nodes should be encompasses if the tumour extended close to the nasopharynx or if there are metastatic neck nodes from an ethmoidal carcinoma.
	In the case it was been resected the margin should encompass all the initial GTV including the dura or the dural graft.			
Maxillary		The inferior border of the maxilla and the hard palate but should encompass a 10-mm margin around the initial GTV.	Medial aspect should be the nasal septum, unless violation of midline structures occurs.	The pterygopalatine and the infratemporal fossa should be included, paying special attention to encompass the masticator space and the infraorbital fissure.

Planning remarks

1. In regions where the GTV is flanked by anatomic barriers (i.e. intact bone), no margin is usually added. In regions where GTV involves compartments enclosed by bone (i.e. maxillary sinus), the whole compartment is contoured as CTV1.
2. When the tumour is in proximity of radiologically defined spaces known to poorly resist to invasion (i.e. masticator or para-pharyngeal spaces) either the entire space or at least part of it should be contoured as CTV2/3. The former is usually reserved for the part close/next to the tumour while the latter for the remaining part.
3. Similarly, when the tumour is abutting the medial wall of the orbit or there is minimal orbital invasion, the medial part (including the rectus medialis muscle) of the orbit has to be included into the CTV2/3.
4. The choice of the regions at risk for microscopic disease depends on the location of the primary tumour, its extension/stage, and pathology. In general, the palate, alveolar ridge, nasal cavity, and the nasopharynx are included in the CTV2/3 in case of maxillary sinus tumours, and the medial orbit in maxillary/ethmoid-sinus cancers. The pterygopalatine fossa and infratemporal fossa, frequently at risk of subclinical disease, are included in the CTV2/LR. Superior lesions require CTV extension to the sphenoid sinus and foramen rotundum at the base of skull to accommodate potential involvement of the maxillary nerve.
5. If MRI suggests neural involvement, the CTV2/3 should be extended to include the cavernous sinus. For lesions in the upper nasal cavity and ethmoid sinuses, the cribriform plate and a rim of the frontal lobe are included in the CTV2/LR. The anterior cranial fossa is included in case of intracranial extension [16].
6. As a general rule, after induction CT, the original location of the tumour (and not the residual) represents the CTV1.
7. In the postoperative setting, CTV2 consists of the resection cavity plus a variable margin according to the principles of a “compartment-related CTV” as described above. All surgically violated regions should be included in the lowest dose level CTV.
8. In case of adenoid cystic carcinoma, attention should be paid to the neural spread and, hence, RT volumes must encompass the afferent and efferent local nerves up to the skull base.
9. Esthesioneuroblastomas arise in the superior nasal cavity and, even at early stages, tend to invade the cribriform plate and anterior cranial fossa, and therefore, these regions should be encompassed in the target volume.

*Lymphnode-level contour*

1. Lymph node metastases are unusual, thus elective treatment of the neck is not mandatory (excluded in the cases of esthesioneuroblastomas, high-grade/high-stage SCC or whenever the next anatomical regions (e.g. nasopharynx, cheek, gingiva or alveolus) are involved.
2. Lymphatic drainage from the paranasal sinuses is to the retropharyngeal, submandibular, and jugodigastric nodes [57]. Cervical metastases are below 10% at presentation thus not justifying elective dissection. For the same reason lymphnodes are usually not included in any CTV unless involved or at risk. The risk of involvement usually depends on the location of the primary tumour and the pathology. Adenocarcinomas and sinonasal undifferentiated cancers (SNUC) rarely spread to lymph nodes; some Authors claim prophylactic neck treatment for esthesioneuroblastomas [150] or SCC of the maxillary sinus [151,152].
3. Once prophylactic neck treatment is indicated, the side of the neck and the levels to be contoured depend on both the location of the tumour and its pathology. For maxillary sinus, levels IB and II represent the first echelon; for esthesioneuroblastomas, *a comprehensive ne*ck irradiation (levels IB-V) is the rule. Bilateral retropharyngeal nodes irradiation should be considered.

Planning remarks

1. After CTV to PTV expansion, overlap between PTV and OAR is frequent and optimal target coverage is often challenging.
2. In general planning priorities are set to avoid a risk of late toxicity >10% on selected OARs as brain, brainstem, chiasm and optic nerves.In the planning process, any attempt should be made to maintain coverage of CTV1 while compromising the coverage of PTV in order to allow minor variations in selected OAR irradiation (i.e. V95% of CTV1 is >95% while PTV1 coverage is 93% and optic nerve Dmax is 59.9 Gy).
3. Proton Beam Therapy has shown good results and acceptable toxicity especially in locally advanced inoperable paranasal tumours [153,154].

### Salivary glands

*Target definition remarks*

Salivary gland tumours are usually treated with surgery. Curative RT alone is an option (even if the odds’ local control is relatively low) for patients with technically unresectable disease or inoperable for medical reasons or who refuse surgery.

Risk factors that support postoperative RT include T3/T4 disease and or any of the following [155,156]:

- Incomplete or close resection margins
- High grade histology
- Recurrent disease
- Peri-neural invasion
- Nodal involvement

### Pathology remarks

Salivary gland tumours include a variety of both benign and malignant tumours that can involve both minor and major salivary glands. In major salivary glands the most frequent benign tumour is pleomorphic adenoma, whereas the most frequent malignant hystology is mucoepidermoid carcinoma; adenoid cystic carcinoma is the most frequent malignant tumour in minor salivary glands. Diagnosis is often difficult and experienced pathologist is needed to interpret fine needle aspiration cytology. Table 19 summarizes the most important pathology variants and the role of RT:

**Table 19:** **Summary of the most important pathology variants in salivary gland tumours**

**Category**	**Variant**	**RT**
Benign mixed tumors	i.e. pleomorphic adenoma	Only for recognized tumour spill; transformation into malignant; recurrent (controversial)
Malignant, low grade	Acinic cell carcinoma	Only for incomplete resection (i.e. close to CN VII), positive or close resection margins; capsule rupture; recurrent
	Mucoepidermoid carcinoma	
Malignant high grade	Mucoepidermoid carcinoma	Always postop RT except in adenoid cystic carcinoma and mucoepdermoid in absence of risk factors.
	Adenocarcinoma	
	SCC	
	Malignant mixed	
	Adenoid cystic	

External beam RT with photons is rarely a curative option since local control in malignant salivary gland tumours is dose dependent. Considering that high doses are needed to achieve local control, most malignant salivary gland tumours would probably benefit from the use of particle therapy. However, although locoregional control after neutron therapy seems to be higher than after photon irradiation, severe late toxicity has been observed while OS was equal. Promising results were found in a dose escalation study in locally advanced adenoid cystic carcinoma. Patients were treated with stereotactically guided or photon IMRT (45-54Gy) and a carbon ion boost of 18 GyE. The results are similar to those obtained with neutrons alone but with lower late toxicity profile [157].

*Dose/fractionation remarks*

Table 20 summarizes the most commonly used postoperative fractionation regimens:

**Table 20:** **Commonly used postoperative regimens**

	**D (Gy)**	**d (Gy)**	**fxs**	**OTT (wks)**	**Comment**
CTV2	66	2	33	6.5	[155]
	63	1.8	35	7	
CTV3	60	2	30	6	[155]
	54	1.8	30	6	
	58.1	1.66	35	7	
	59.4	1.8	33	6.5	

In general the radiosensitivity of salivary gland tumours depends on their pathology; doses for SCC are similar to SCC of other districts; for adenoid cystic doses in the order of 66 Gy and 60 Gy (at 2 Gy per fr) are recommended for CTV2 and CTV3, respectively [155] A dose of 60 Gy for postoperative treatment of high grade tumours has been suggested also by Chen et al. [158].

**Table 21:** **Suggested volumes (surgical bed) at high risk**

**Site**	**Anterior**	**Lateral**	**Medial**	**Posterior**
Parotid surgical bed	Masseter muscle	Soft tissue of neck	Styloid process	Mastoid bone
Submandibular surgical bed	Follow clips if leaved by surgeon otherwise and use the contralateral submandibular gland as a guide.			

*Primary tumour contour*

1. A bolus over the scar region is indicated in the case of skin invasion, for superficially located tumours of the parotid or submandibular glands and/or in case of tumour spillage/ capsular rupture.
2. Adenoid cystic tumours have the distinct propensity for perineural infiltration (facial, trigeminal, hypoglossal, lingual nerve) and skull base involvement; in case of ‘named’ nerve invasion, CTV3 must track the course of the nerve as back as to its entry in the skull (base of skull). The addition of concomitant adjuvant CT is still investigational.
3. The CTV is a function of the location of the tumour (Table 21), its extension and histological risk factors; for low grade tumours, only the tumour site (i.e. parotid bed) is at risk; for high grade tumours, CTV2 includes the initial site of disease, while CTV3 should cover the whole surgical bed.
4. For parotid gland tumours, a document online describes target volume definition of the parotid bed and indications for ipsilateral neck irradiation for patients entering the COSTAR trial (COchlear Sparing Therapy And Conventional Radiation: A Multicentre Randomised Study Of Cochlear Sparing Intensity Modulated RT Versus Conventional RT In Patients With Parotid Tumours)

http://aktinotherapeutis.gr/wp-content/uploads/doctors/COSTAR_Outlining_Guidelines.pdf

4. The parotid gland extends from the zygomatic arch superiorly to beyond the lower border of the mandible inferiorly. Posteriorly, the gland dips in the space between the mandible and the mastoid, with the adjoining external auditory meatus intimately surrounded by the gland in its free borders, that is, anteriorly and inferiorly. On a deeper plane, the parotid is related to the styloid process and the muscles connected to it (Figure 4). Local infiltration of tissues adjacent to the parotid gland is the main pattern of spread. This follows the anatomical borders of the parotid gland and in cases of perineural invasion, the facial nerve to the stylomastoid foramina.

**Figure 4:**
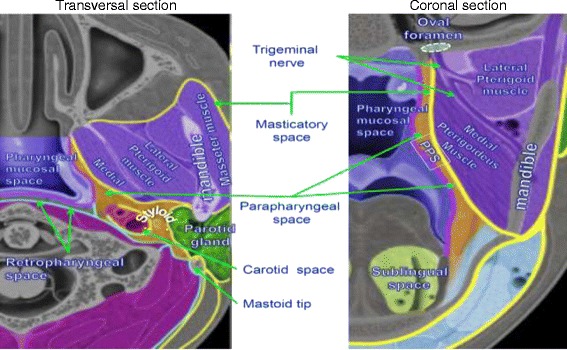
**Parapharyngeal space (transversal and coronal sections).**
5. CTV2 for parotid tumours includes: the parotid bed and any adjacent tissues at risk of microscopic spread.
  - Areas at risk of soft tissue extension are: infratemporal fossa, parapharyngeal space, masseter and digastric muscle, skin.
  - Bone infiltration-at risk areas include: lateral part of the floor of middle cranial fossa, neck of mandible, external auditory meatus, inferior surface of styloid process.

*Lymphnode-level contour*

1. Elective nodal irradiation should be considered in any of the following [158]:
  - High grade tumours
  - T3/T4 stage
  - Histological subtype
    i. Squamous cell carcinomas
    ii. Adenocarcinomas
    iii. Undifferentiated carcinomas,
    iv. High grade mucoepidermoid carcinomas
    v. Salivary duct carcinomas
2. In case of risk of subclinical disease, if the primary tumour is dissected, the lymph node levels at risk should be dissected as well (surgical/pathological staging of the neck is usually preferred to elective treatment of undissected neck).
3. After surgery, planning should avoid underdosing of the skin especially when capsular rupture is present (bolus over the scar).
4. Levels Ib, IIa IIb, RP ipsilateral nodal stations (commonly in tumor bed) should be included in CTV2 for parotid tumors.
5. Levels III, IV and V ipsilateral nodal stations should be included in CTV3 for parotid tumors when high grade features are present, T3-T4, and the histological type listed in point 1 if neck is undissected [159,160].
6. There is no indication for bilateral elective neck treatment.

### Neck metastases from unknown primary (cTx)

*General remarks*

Metastatic cervical nodes of unknown primary origin represent a very heterogeneous entity. Squamous cell carcinoma is the more frequent histological type, followed by adenocarcinoma and undifferentiated carcinoma.

When pathology is different from squamous cell carcinoma (eg melanoma, lymphoma, adenocarcinoma), a primary arising in the chest, thyroid or salivary gland cancer should be suspected and treatment should be planned accordingly (the following guidelines would not apply).

Here we consider only SCC or undifferentiated carcinoma.

There are some correlations between the location of the node and the primary site:

1. superficial parotid: skin;
2. level Ib: oral cavity;
3. level Va: NPC;
4. level IV: chest (or abdomen)

More than 50% of primaries are discovered by physical examination and assessment under anaesthesia conducted by experienced ENTs (this data refers to pre-PET era) [161]; 50-80% of primaries are found in the oropharynx; there is a strong correlation between HPV, cystic nodes and oropharyngeal primary [162], but up to 25% of patients may have primary disease below the clavicles (lungs). EBV testing may help determine possible primary location. There is no advantage for repeated Direct Laryngoscopy if negative initially [163].

More than FNA (e.g. incisional/excisional biopsy) is considered ‘neck violation’ and should be avoided. Injudicious removal of a metastatic node causes surgical scarring which may prevent or preclude subsequent surgical dissection of the lymph node bearing area. Above all, this procedure may delay the proper treatment.

Unilateral or bilateral tonsillectomy should be performed including excision of eventual remnant tonsillar tissue. In the absence of panendoscopic detectable lesions, ipsilateral tonsillectomy can discover carcinoma in about a quarter of the patients [164]. If physical examination shows mucosal abnormalities the detection rate rises to 40%. It is still controversial if tonsillectomy should be bilateral or unilateral. In favour of bilateral tonsillectomy, some Authors report that the rate of contralateral spread of metastatic cancer from occult tonsil lesions may approach 10%. A more convincing reason is to avoid asymmetric FDG uptake in the oropharyngeal region by removing only one tonsil that may confound follow up studies. For these reasons, bilateral tonsillectomy is recommended as a routine step in the search for the occult primary in patients presenting with cervical metastasis of SCC and palatine tonsils intact [165]. The discovery of the primary lesion may help the radiation oncologist to better define volumes to be treated and their respective dose. This ultimately would help to increase the odds of cure and minimize the risk of side effects. If physical examination and other imaging modalities are negative, PET may discover new primaries in an additional 25% of patients (5-73%), nodes in ≈ 15% and distant metastases in ≈ 10% [166]. Because FDG-PET detects increased metabolic activity, inflammation induced by biopsies may contribute to false-positive results if PET is obtained after direct laryngoscopy/biopsy [167].

*General treatment strategy*

There are 2 main clinical scenarios:

1. MACROSCOPIC TUMOUR RESIDUAL AFTER INITIAL EVALUATION: Surgery vs RT ± CT

In favour of neck surgery stand the following remarks:

1. If the patient most likely needs surgery after RT or chemoRT (i.e. bulky node);
2. If the patient may be potentially cured by surgery alone (i.e. small <3 cm/single node);
3. Surgery permits concomitant teeth extractions and Direct Laringoscopy;
4. Surgery allows for a better staging of the neck and pathology of node level involvement;
5. If more tissue is required and pathology of nodal diffusion is uncertain.

However, surgery may add extra morbidity especially if subsequent RT is necessary.

In favour of RT ± CT stand the following remarks:

1. If RT or CT-RT are potentially curative in patient that would need postoperative RT anyhow (i.e. multiple small nodes);
2. The use of RT or CT-RT without surgery on the neck allows for a better oxygenation of the potential mucosal site of primary;
3. It permits better evaluation of tumour response (especially if CT is used); leaving salvage surgery for non-responders.

2. NO RESIDUAL MACROSCOPIC DISEASE AFTER INITIAL EVALUATION

If pN1 *AND* no ECE (AND no previous neck violation), observation can be appropriate; otherwise, if pN > 1 or ECE or neck violation, postoperative RT should be recommended.

*Target definition remarks*

- CTV1 or high disease volume that encompasses the gross tumour volume in the neck if present with a margin;
- CTV2 that typically includes the putative primary site(s) considered at high risk of containing microscopic disease; moreover, after surgery, this would include the site of dissected positive lymph nodes;
- CTV3 includes the nodal stations that do not contain positive or suspicious nodes after appropriate imaging; moreover, it would include the putative primary site(s) considered at low risk of containing microscopic disease.

*Dose/fractionation remarks*

Tables 22 and 23 show the commonly used fractionation regimens in definitive (macroscopic nodal disease present) and in postoperative settings:

**Table 22:** **Suggested fractionation regimens for Unknown Primary (macroscopic disease present)**

	**D (Gy)**	**d (Gy)**	**Fxs**	**OTT (wks)**	**Comment**
CTV1	70	2	35	7	
	66	2.2	30	6	[168]
CTV2	63	1.8	35	7	
CTV3	58.1	1.66	35	7	
	54	1.8	30	6	[168]

**Table 23:** **Suggested fractionation regimens for Unknown Primary (postoperative setting)**

	**D (Gy)**	**d (Gy)**	**fxs**	**OTT (wks)**	**Comment**
CTV2	60-64	2	30-32	6	(64 Gy if ECE+) [168]
	63	1.8	30	6	
CTV3	58.1	1.66	35	7	
	54	1.8	30	6	

*Primary-tumour contour*

1. According to classical teaching, the putative primary tumour may be located in the pharyngeal axis and thus the mucosa of the whole pharynx (nasopharynx, oropharynx and hypopharynx) as well as the larynx would be included in the subclinical dose level; the oral cavity should NOT be routinely included. The extent of the pharynx to be irradiated must be determined on a case-by-case basis [169]:
  - Irradiation of oropharynx alone might be sufficient for an HPV+ patient.
  - Irradiation of nasopharynx alone might be sufficient for an EBV+.
2. The larynx and hypopharynx are considered at low risk if levels III and IV are not involved *and* PET/CT is negative *and* level V is positive and pathology is suspicious for a nasopharyngeal primary *or* the tumour stains for HPV and/or EBV. In these cases, the dose to the larynx can be lowered to CTV3 or even dropped from any CTV [168,170-173].

*Lymphnode-level contour*

1. Traditionally, all neck levels ipsilateral to the positive nodal disease are included in the target volume.
2. With regard to the bilateral neck irradiation, currently there are two points of view:
  - Considering the morbidity induced by extensive irradiation, some Authors consider irradiation of the contralateral neck indicated for a defined subset of patients, e.g., those with bilateral nodal metastases, extensive unilateral involvement with regard to number and levels of nodal metastases (cN2b+), unfavourable grading [164].
  - Other Authors, reporting local control rates with median neck relapse rate ranging from 31 to 63% after unilateral RT group, compared with a median neck relapse rate ranging from 8 to 49% in bilateral and pharyngeal mucosa RT group, advocate bilateral neck irradiation [174]. In this case contralateral neck levels II-IV and the retropharyngeal nodes could be included in CTV3 [169].
3. Bilateral retropharyngeal nodes should be included.
4. Ipsilateral level Ib can be excluded if levels II and III are uninvolved.
